# FOXG1 drives transcriptomic networks to specify principal neuron subtypes during the development of the medial pallium

**DOI:** 10.1126/sciadv.ade2441

**Published:** 2023-02-15

**Authors:** Ru Ba, Lin Yang, Baoshen Zhang, Pengfei Jiang, Zhipeng Ding, Xue Zhou, Zhengang Yang, Chunjie Zhao

**Affiliations:** ^1^ Key Laboratory of Developmental Genes and Human Diseases, Ministry of Education, School of Medicine, Southeast University, Nanjing 210009, China.; ^2^ State Key Laboratory of Medical Neurobiology, Institutes of Brain Science, MOE Frontier Research Center for Brain Science, Fudan University, Shanghai 200032, P.R. China.

## Abstract

The medial pallium (MP) is the major forebrain region underlying learning and memory, spatial navigation, and emotion; however, the mechanisms underlying the specification of its principal neuron subtypes remain largely unexplored. Here, by postmitotic deletion of FOXG1 (a transcription factor linked to autism spectrum disorders and *FOXG1* syndrome) and single-cell RNA sequencing of E17.5 MP in mice, we found that FOXG1 controls the specification of upper-layer retrosplenial cortical pyramidal neurons [RSC-PyNs (UL)], subiculum PyNs (SubC-PyNs), CA1-PyNs, CA3-PyNs, and dentate gyrus granule cells (DG-GCs) in the MP. We uncovered subtype-specific and subtype-shared FOXG1-regulated transcriptomic networks orchestrating MP neuron specification. We further demonstrated that FOXG1 transcriptionally represses *Zbtb20*, *Prox1*, and *Epha4* to prevent CA3-PyN and DG-GC identities during the specification of RSC-PyNs (UL) and SubC-PyNs; FOXG1 directly activates *Nr4a2* to promote SubC-PyN identity. We showed that TBR1, controlled by FOXG1 during CA1-PyN specification, was down-regulated. Thus, our study illuminates MP principal neuron subtype specification and related neuropathogenesis.

## Teaser

Transcriptomic mechanisms orchestrate specification of principal neurons of the archicortex and the intermediate cortex.

## INTRODUCTION

In mammals, the medial pallium (MP) of the telencephalon comprises the archicortex, which consists of the hippocampal CA1-CA3, the dentate gyrus (DG) and the subiculum (SubC), and the “intermediate” retrosplenial cortex (RSC), which is located between the archicortex and neocortex. These components of the MP are anatomically and functionally interconnected, underpinning a range of cognitive functions, including episodic and spatial memory, navigation, and imagination. DG granule cells (DG-GCs) receive a vast amount of highly processed multimodal sensory information from the entorhinal cortex and are conveyed to CA3 pyramidal neurons (CA3-PyNs); the latter connect to CA1-PyNs by Schaffer collaterals, thus forming the so-called DG-CA3-CA1 trisynaptic circuitry. The SubC is a patchwork of the hippocampus positioned in the dorsal part of the CA1. SubC-PyNs act as a primary output, receiving inputs from CA1-PyNs and conveying hippocampal signals to various cortical and subcortical areas (*1*). The RSC is notably connected with the SubC, the laterodorsal thalamic nuclei and the entorhinal cortex (*2*). Connections between RSC-PyNs and these structures are reciprocal, suggesting that RSC-PyNs may serve as a site of information storage (*3*). It is now evident that the MP is consistently compromised in many neurological and psychiatric diseases. Understanding how principal neuron subtypes are specified will certainly expand our knowledge of MP development and help resolve the etiopathology of numerous neurological disorders.

MP principal neurons emerge from the ventricular zone in partially overlapping generation waves. The generation peak time of CA1-PyNs, CA3-PyNs, and SubC-PyNs is around embryonic day 14 (E14) to E15, and the majority of RSC-PyNs are born during E12 to E15 (*4*). After exiting the cell cycle, newborn neurons proceed with postmitotic specification to acquire their final characteristics. This acquisition is coordinated by the sequential activation/repression of gene expression programs that are mediated by transcription factor (TF) networks. Great progress has been made in characterizing the specification of neocortical neurons. However, less attention has been given to principal neuron specification of the archicortex and the intermediate cortex (i.e., MP), especially RSC-PyNs, SubC-PyNs, and CA3-PyNs, despite their key function in cognition. Recently, studies have shown that the TF prospero homeobox 1 (PROX1) specifies a DG-GC fate over a CA3-PyN fate (*5*). Loss of *Zbtb20* causes a progressive ventral displacement and molecular misspecification of all MP domains—RSC into SubC, SubC into CA1, CA1 into CA2, and CA2 into CA3, accompanied with reduced size of CA1, CA3, and DG (*6*). Conversely, ectopic expression of *Zbtb20* forces SubC-PyNs and RSC-PyNs [upper layer (UL)] into a hippocampus-like identity (*7*). The TF special AT-rich sequence binding protein 2 (SATB2) was established to shape RSC-PyNs (UL) (*8*). Although there have been true breakthrough advances in our understanding, much remains unknown about the transcriptomic networks that regulate the postmitotic specification of principal neuron subtypes in the developing MP.

The Forkhead-box TF FOXG1 has been demonstrated to control a variety of developmental processes ranging from dorsal-ventral pattern formation of the telencephalon and cell fate determination to cortical circuit specialization by exerting both repressive and activating functions (*9*, *10*). However, no study has examined the contribution of FOXG1 to postmitotic cell type specificity in the MP or the downstream targets of FOXG1 at single-cell resolution. In the present study, we generated mice with deletion of *Foxg1* in postmitotic neurons by crossing a *Nex-Cre* line with *Foxg1^fl/fl^* mice (*11*). We show that loss of *Foxg1* leads to impaired specification of MP principal neuron subtypes. By single-cell RNA sequencing (scRNA-seq) using E17.5 MP, we characterized the molecular profiles of principal neuron subtypes in the developing MP in control and *Foxg1* conditional knockout (cKO) mice. We identified subtype-specific and subtype-shared FOXG1-driven gene networks. In support of the pleiotropic function of FOXG1, using chromatin immunoprecipitation–quantitative polymerase chain reaction (ChIP-qPCR) and luciferase assays combined with in utero electroporation (IUE) methods, we demonstrated the transcriptional repression of FOXG1 on *Zbtb20*, *Prox1*, and *Epha4* to prohibit the identities of CA3-PyNs and DG-GCs and the activation of *Nr4a2* to promote SubC-PyN identity. Our study supports the notion that FOXG1 orchestrates the postmitotic specification of MP principal neuron subtypes via both subtype-specific and subtype-shared transcriptional programs.

## RESULTS

### Disrupted identities of MP principal neuron subtypes after postmitotic deletion of *Foxg1*

**Fig. 1.**
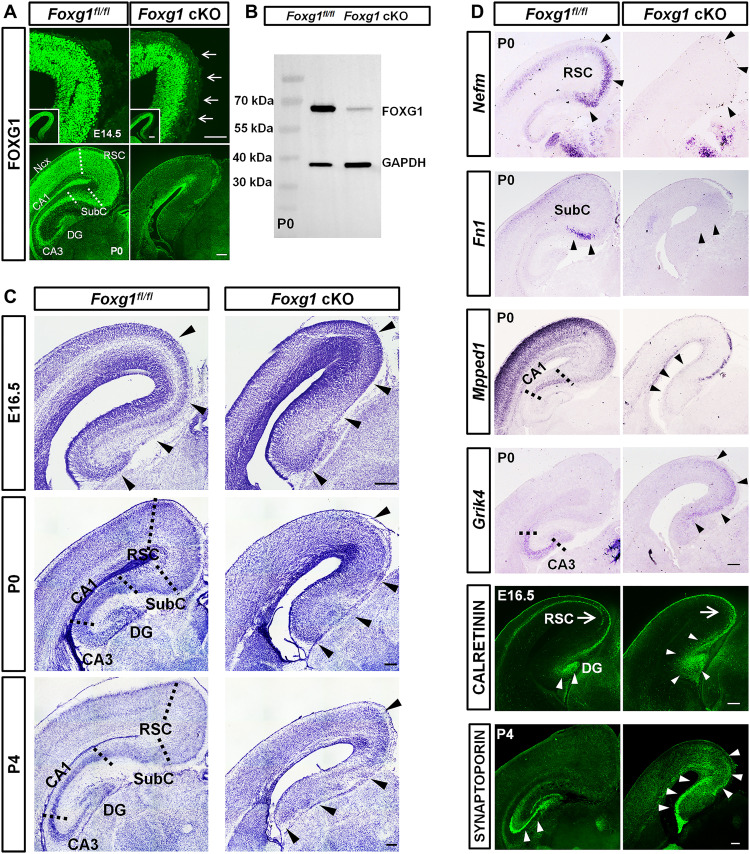
Postmitotic deletion of *Foxg1* disrupts MP morphogenesis and subtype identity. (**A**) Immunostaining of anti-FOXG1 at E14.5 and P0 showing that FOXG1 was efficiently disrupted in postmitotic neurons in the *Foxg1* cKO MP. (**B**) Western blotting of P0 MP extracts showing that FOXG1 was efficiently disrupted in *Foxg1* cKO mice. (**C**) Nissl staining of coronal sections of E16.5, P0, and P4 brains showing that the typical features of the RSC, SubC, CA1-CA3, and DG disappeared in *Foxg1* cKO mice. (**D**) In situ hybridization of *Nefm*, *Fn1*, *Epha4*, *Mpped1*, and *Grik4* at P0 and immunostaining against CALRETININ and SYNAPTOPORIN at E16.5 and P4, respectively, showing the loss of the RSC UL and SubC and the notable expansion of CA3 and the DG in *Foxg1* cKO mice. Nex, neocortex; RSC, retrospenial cortex; SubC, subiculum; Hipp, hippocampus; DG, dentate gyrus; GAPDH, glyceraldehyde-3-phosphate dehydrogenase. Scale bars, 200 μm (A, C, and D) and 50 μm (the magnification of A).

To explore the contribution of FOXG1 to MP development, we knocked out *Foxg1* by crossing *Foxg1^fl/fl^* mice with *Nex-Cre* mice in which CRE-mediated recombination occurs in postmitotic neurons from E11.5 onward (*Nex-Cre*; *Foxg1^fl/fl^* cKO, hereafter designated as “*Foxg1* cKO”). Supporting efficient deletion, immunostaining of anti-FOXG1 at E14.5 and postnatal day 0 (P0) showed that FOXG1 was highly expressed in the MP postmitotic neurons of control mice (*Foxg1^fl/fl^*, hereafter designated as “control mice”) but was undetectable in postmitotic MP neurons of the *Foxg1* cKO mice (Fig. 1A). Immunoblotting of *Foxg1* cKO P0 MP extracts revealed a substantially reduced FOXG1 level, confirming efficient disruption in the MP (Fig. 1B).

We conducted Nissl staining to examine MP morphogenesis at E16.5, a time point at which MP formation becomes evident: In the control MP, densely packed neurons appeared in the RSC and the incipient hippocampal CA regions, whereas no packed neurons were observed in the *Foxg1* cKO MP (Fig. 1C). At P0, neurons dispersed throughout the entire *Foxg1* cKO MP, and there were no obvious borders between the RSC and the SubC or between the SubC and the CA1 compared to the evident borders in the control MP (Fig. 1C and fig. S1). For P4 *Foxg1* cKO mice, the morphological abnormalities were more severe than at P0, and there was not a typical compact neuron layer in the CA1-CA3, a less dense neuron layer in the SubC, or a typical V-like packing of neurons in the DG, whereas each distinct region exhibited its expected characteristic features in the control MP (Fig. 1C). These results demonstrate that loss of *Foxg1* results in severe morphological abnormalities in the MP.

To assess the identities of neurons dispersed in the MP after postmitotic deletion of *Foxg1*, we next used in situ hybridization of P0 brain sections to examine the levels of a set of genes known to be specifically expressed in distinct subtypes of MP principal neurons. In the control MP, *Nefm* was intensely expressed in the RSC-PyNs (UL), and *Fibronectin 1* (*Fn1*) was strongly expressed in the SubC-PyNs; no *Nefm* or *Fn1* was detected in the *Foxg1* cKO MP (Fig. 1D). We then assessed *Mpped1*, a commonly used marker gene for CA1-PyNs (*12*). A signal for *Mpped1* was detected in the control CA1 region, whereas no *Mpped1*^+^ CA1-PyNs were detected in the *Foxg1* cKO mice (Fig. 1D). CA3-PyNs of control mice had a clear *Grik4* signal, whereas the *Grik4* signal was obviously dorsally expanded toward the CA1, SubC, and RSC in the *Foxg1* cKO mice (Fig. 1D). We also conducted immunostaining against CALRETNIN, a calcium-binding protein that marks immature DG-GCs (*13*), and found that in control mice, CALRETNIN^+^ neurons were limited to the primary DG at E16.5, whereas these were spread extensively throughout the *Foxg1* cKO MP (Fig. 1D). Together, these altered expression patterns of marker genes suggest dysregulation of the identities of principal neuron subtypes in the *Foxg1* cKO MP.

The observed impairment of neuron identities resulting from cKO of *Foxg1* would be expected to manifest in projection phenotypes. Immunostaining against CALRETNIN also displayed a complete loss of corticofugal tracts projected from the RSC-PyNs in the *Foxg1* cKO mice (Fig. 1D). Consistent with the expansion of the DG-GCs, anti-SYNAPTOPORIN staining at P4 revealed that the *Foxg1* cKO mice had thicker mossy fibers than the controls, and these fibers aberrantly invaded the SubC and RSC regions (Fig. 1D). These findings collectively demonstrate that FOXG1 controls the specification of principal neuron subtypes of the MP.

### *Foxg1* KO dysregulates genes involved in regionalization, cell fate commitment, and neuronal differentiation

**Fig. 2.**
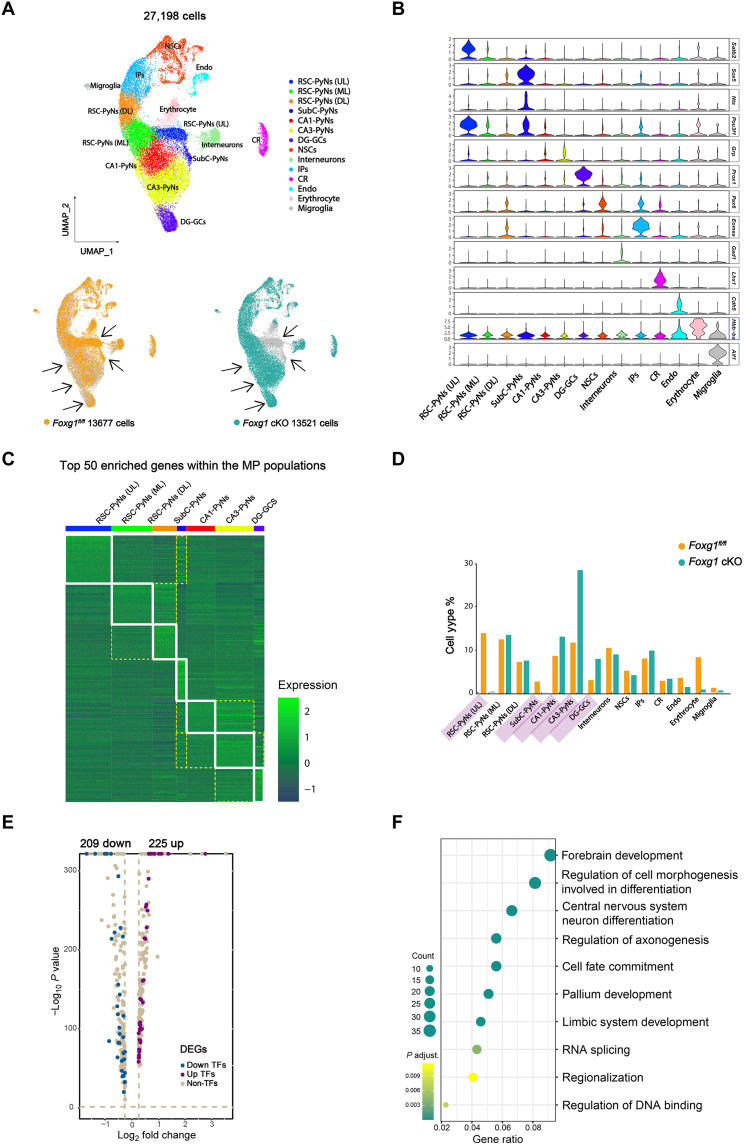
Postmitotic deletion of *Foxg1* dysregulated genes involved in regionalization, cell fate commitment, and neuronal differentiation. (**A**) UMAP plot showing 27,198 cells from the MP of *Foxg1*-deficient and control mice at E17.5, colored by cluster annotation. Cell type annotation is overlaid to identify the major cell type represented by each cluster (14 total clusters). UMAP plots of cells from the left color coded to identify each cell by genotype. Arrows indicate populations of RSC-PyNs (UL), SubC-PyNs, CA1-PyNs, CA3-PyNs, and DG-GCs. (**B**) Identification of cell subtypes based on known marker genes. (**C**) Heatmap depicting the expression of the top 50 enriched genes in each MP principal neuron cluster. Each column represents a cell, and each row represents a gene. Genes are grouped on the basis of specific cluster expression, and cells are ordered on the basis of clustering. The expression is normalized by gene. For all significantly enriched genes per cell type, see table S1. (**D**) Bar plots showing the percentages of subtype neurons among the total analyzed cells between the two genotypes. (**E**) Volcano plots showing MP-DEGs between *Foxg1* cKO and control mice. (**F**) Bubble chart showing GO terms enriched for MP-DEGs between *Foxg1* cKO and control mice. PyNs, pyramidal neurons; GC, granule neurons; UL, upper layer; ML, middle layer; DL, deep layer; NSCs, neural stem cells; IPs, intermediate progenitor cells; CR, Cajal-Retzius; Endo, endothelial cells.

To explore how FOXG1 influences the transcriptome to specify neuron subtypes, we conducted scRNA-seq of 27,198 cells sampled from the control and *Foxg1* cKO MPs at E17.5, a time point at which the majority of MP principal neurons have already been born and undergone postmitotic specification (*14*). We detected an average of 4053 unique molecular identifiers and 1873 genes (median = 1580) per cell. Uniform manifold approximation and projection (UMAP) was then performed in these cells using Seurat v4.04 (*15*), which resulted in 14 distinct clusters (Fig. 2A), which were annotated as known cell types based on previously reported marker genes (Fig. 2B). We performed Wilcoxon rank sum tests within each cluster pair to identify the top 50 genes with enriched expression in MP principal neuron subtypes (adjusted *P* value < 0.05, |log_2_ fold change (log_2_FC)| > 0.25), and the control MP subtypes all exhibited unique gene profiles that were different from each other (Fig. 2C and table S1). It should be mentioned that although each MP subtype exhibited its own unique gene profile reflected by its top 50 enriched genes, some genes with enriched expression in one subtype were coexpressed in two or more other subtypes; for example, genes with enriched expression in RSC-PyNs (UL), CA1-PyNs, and CA3-PyNs were coexpressed in Subc-PyNs. CA1-PyNs and CA3-PyNs also shared some gene expression properties (Fig. 2C and table S1).

We next compared the proportions of MP principal neuron subtypes between *Foxg1* cKO and control mice. As expected, the proportion of RSC-PyNs (UL) among total MP cells was sharply decreased in *Foxg1* cKO mice (~0.1% versus ~13.8% in control mice); there was also an obvious reduction in *Foxg1* cKO SubC-PyNs (~0.03% compared ~2.8% in controls) (Fig. 2, A and D). In contrast, the *Foxg1* cKO MPs showed significantly increased proportions of CA3-PyNs (28.3% versus 11.7% in controls) and DG-GCs (8% versus 3.15% in controls) (Fig. 2, A and D). Unexpectedly, although we did not detect *Mpped1* signal in the *Foxg1* cKO CA1 by in situ hybridization (Fig. 1D), the proportion of *Foxg1* cKO CA1-PyNs was increased to ~13.1% among total MP cells, a 1.5-fold increase over the control ~8.68% (Fig. 2D). We then carefully analyzed the scRNA-seq data and found that the expression of typical markers for CA1-PyNs (e.g., *Mpped1*, *Pou3f1*, and *Cck*) was not detectable (Fig. 1D and fig. S2), coincident with the in situ hybridization analysis of P0 brain sections, but there was an obvious up-regulation of the expression of marker genes (e.g., *Zbtb20* and *Nrp2*) coexpressed with CA3-PyNs, a subtype known to share similar morphology, functions, and electrical properties with CA1-PyNs (fig. S2). Thus, the increased proportion of *Foxg1* cKO CA1-PyNs as indicated by the scRNA-seq data appears to be a consequence of the up-regulation of marker genes coexpressed between CA1-PyNs and CA3-PyNs. The proportions of other cell subtypes in which *Foxg1* was not deleted, including interneurons, progenitors/neural stem cells, and microglial cells, did not differ between the two genotypes (Fig. 2, A and D). No marked changes were detected in RSC-PyNs (middle layer) or RSC-PyNs (deep layer) (Fig. 2, A and D).

We next performed a differential gene expression analysis among MP principal neurons between the two genotypes and identified a total of 434 differentially expressed genes (DEGs), with 225 up-regulated and 209 down-regulated genes after *Foxg1* deletion (Fig. 2E and table S2). Gene ontology (GO) analysis of the DEGs supported roles for FOXG1 associated with the terms “forebrain development,” “neuron morphogenesis and differentiation,” “cell fate commitment,” “regionalization,” “axonogenesis,” and “DNA binding and RNA splicing” (Fig. 2F). Collectively, these findings demonstrate that conditional *Foxg1* KO affects the expression of a gene network that is involved in postmitotic neuron differentiation during MP development.

### Identification of candidate FOXG1 targets genes in the developing MP

**Fig. 3.**
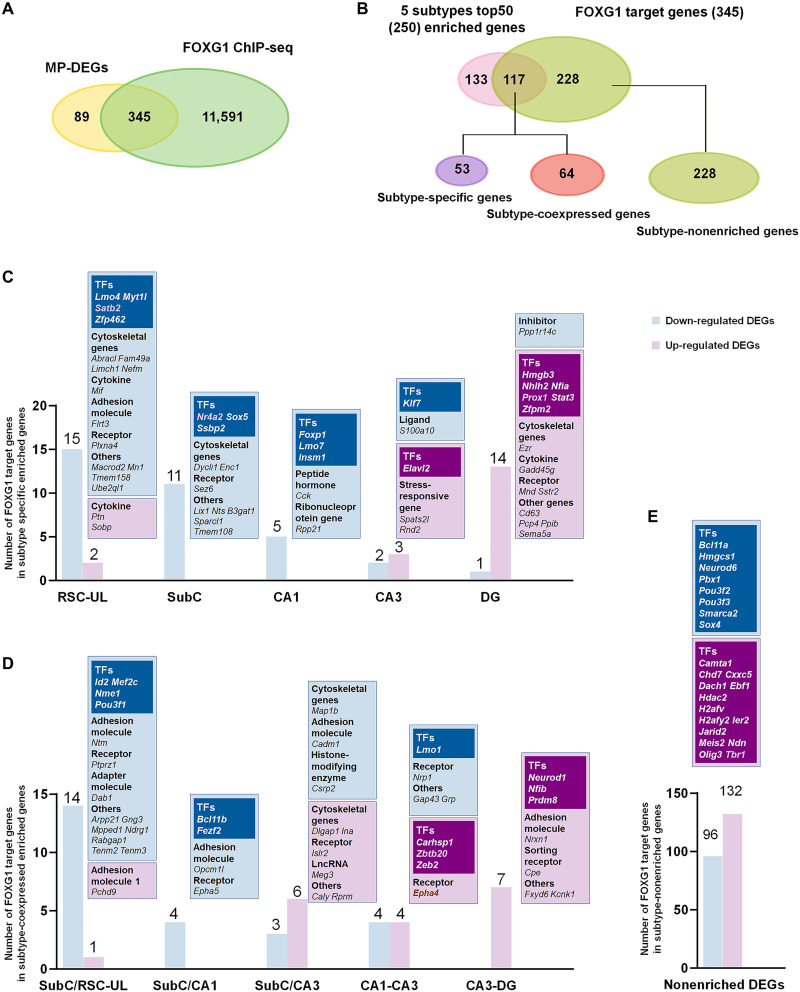
Identification of FOXG1-driven transcriptomic networks. (**A**) Pie plot showing overlapping genes between DEGs among MP principal neurons (MP-DEGs) and FOXG1 ChIP-seq. (**B**) Pie plot showing overlapping genes between 250 enriched genes in the MP neuron subtype and candidate FOXG1 target genes, which sorted into three categories: 53 subtype-specific genes, 64 subtype-coexpressed genes, and 228 subtype-nonenriched genes. (**C**) Identification of FOXG1-driven subtype-specific genes. (**D**) Identification of FOXG1-driven subtype-coexpressed genes. (**E**) Identification of FOXG1-driven subtype-nonenriched genes.

We next aimed to investigate the influence of FOXG1 on gene regulatory networks to specify MP neuron subtypes. We assessed the 434 DEGs by joint analysis with publicly available ChIP sequencing (ChIP-seq) data for FOXG1 from wild-type embryonic dorsal cortices [Gene Expression Omnibus (GEO) database, GSE96070] (*16*), which identified a total of 345 candidate FOXG1 target genes (Fig. 3A and table S3). Because the acquisition of a neuron subtype identity is presaged by its combinatorial activity of transcription programs, these 345 candidate FOXG1 target genes were then overlaid with a total of 250 genes that were the top 50 enriched genes from each MP subtype (a total of five subtypes) added together. Among 345 candidate FOXG1 targets, 117 overlapped genes were identified as subtype-enriched genes in the MP, and the other 228 represented genes were expressed at relatively low levels in the MP subtypes (Fig. 3B). Next, candidate FOXG1 targets were classified into three categories for further analysis: (i) 53 genes with enriched expression specific to a distinct subtype to which we used the term “subtype-specific genes” to refer, (ii) 64 genes with enriched expression in a distinct subtype but coexpressed in two or more subtypes to which we used the term “subtype-coexpressed genes,” and (iii) 228 genes expressed at lower levels relative to enriched genes in one or more subtypes to which we used the term “subtype-nonenriched genes” to refer (Fig. 3B).

### Characterization of subtype-specific FOXG1-driven transcriptional networks

To investigate FOXG1-driven networks defining each distinct subtype, we first analyzed 53 subtype-specific candidate FOXG1 target genes. For *Foxg1* cKO RSC-PyNs (UL), we identified 15 severely down-regulated genes (Fig. 3C and fig. S3A). Among four TFs (*Satb2*, *Myt1l*, *Lmo4*, and *Zfp462*), *Satb2* is known to be required for RSC-PyN identity (*8*). *Myt1l* was reported to drive fibroblast-to-neuron conversion and to control the switch of neuronal proliferation/differentiation (*17*), indicating its involvement in MP neuron specification. Previous studies have shown that *Lmo4* regulates synaptic plasticity in the adult hippocampus (*18*) and that *Zfp462* is related to anxiety-like behaviors (*19*), suggesting its possible roles in subtype specification and functional maintenance in the MP. A set of genes—including cytoskeletal genes (e.g., *Abracl*, *Fam49a*, *Limch*, and *Nefm*), cytokines (e.g., *Mif*, linked to autism spectrum disorders and epilepsy), cell adhesion molecules (e.g., *Flrt3*), and receptors (e.g., *Plxna4*), which are involved in axon, dendritic development and synaptogenesis (*20*, *21*)—were also identified to be down-regulated after *Foxg1* deletion (Fig. 3C and fig. S3A). These genes may represent potential FOXG1 target genes that shape RSC-PyNs (UL).

We found that 11 SubC-PyN–specific candidate FOXG1 target genes were down-regulated after *Foxg1* deletion (Fig. 3C and fig. S3B). Among three TFs (*Nr4a2*, *Sox5*, and *Ssbp2)*, *Nr4a2* was reported to be required for the specification of SubC-PyNs (*8*). *Sox5* was previously demonstrated to regulate the developmental trajectory of cortical corticofugal neurons (*22*). *Ssbp2* is shown to be associated with adult neurogenesis; SSBP2 interacts specifically with Chip/LIM domain-binding proteins (LDBs) and stabilizes LDBs by preventing their proteasomal degradation, which controls cell fate decisions (*23*). These data suggest that down-regulation of nuclear receptor subfamily 4, group A, member 2(NR4A2), SRY (sex determining region Y)-box 5 (SOX5), and single-stranded DNA binding protein 2 (SSBP2) may contribute to an aberrant transcriptional regulatory network, leading to the loss of SubC-PyN identity in *Foxg1* cKO MPs. Similar to RSC-PyNs (UL), in *Foxg1* cKO SubC-PyNs, we identified down-regulated cytoskeletal genes (e.g., *Enc1* and *Dync1i1*), ligands (e.g., *Nts*), and receptors (e.g., *Sez6*) (Fig. 3C and fig. S3B) related to neuron morphogenesis (*24*–*27*), and these genes may also be essential for the morphogenesis of SubC-PyNs.

The down-regulated CA1-PyN–specific candidate FOXG1 that targets TFs after *Foxg1* deletion included *Lmo7*, *Insm1*, and *Foxp1* (Fig. 3C and fig. S4A). *Lmo7* was reported to be required for proper myoblast differentiation by temporally regulating myogenic differentiation genes (*28*), and *Foxp1* was shown to be required for neuronal differentiation in the neocortex and the hippocampus (*29*). Mutations in *Foxp1* are causative for neurodevelopment disorders such as autism spectrum disorders (*30*). *Insm1* was reported to be involved in the postmitotic differentiation of sensory cells (*31*). We also noticed that the peptide hormone cholecystokinin, which regulates neuronal excitability (*32*), was substantially down-regulated in *Foxg1* cKO CA1-PyNs (Fig. 3C). These dysregulated target genes may underlie the progressive establishment of CA1-PyN characteristics.

In *Foxg1* cKO mice, we identified an up-regulated CA3-PyN–specific TF, *Elavl2* (Fig. 3C and fig. S4B), a candidate gene for schizophrenia, which functions as an RNA binding protein to regulate *Mmp-9* mRNA stabilization in hippocampal neurons for electrical activity (*33*). Another CA3-PyN–specific TF, *Klf7*, which was reported to be required for neuronal morphogenesis and axon guidance in several regions, including the hippocampus and neocortex (*34*), was down-regulated after *Foxg1* deletion (Fig. 3C and fig. S4B). Thus, *Elavl2* and *Klf7* may be involved in shaping CA3-PyN identity.

For DG-GC–specific TFs, *Prox1*, *Stat3*, *Zfpm2*, *Nhlh2*, *Hmgb3*, and *Nfia*, were found to be substantially up-regulated in the absence of *Foxg1* (Fig. 3C and fig. S4C). *Prox1* has been demonstrated to control DG-GC identity. Loss of *Prox1* deprives the signature of gene expression of DG-GCs (*5*). *Stat3* controls hippocampal neuronal maturation and terminal differentiation (*35*), and *Zfpm2* is involved in the specification of corticothalamic neurons during development (*36*). These dysregulated TFs may contribute to the increased DG-GCs in the *Foxg1* cKO MP. Dysregulated cytoskeletal genes (e.g., *Ezr*), cytokines (e.g., *Gadd45g*), ligands (e.g., *Sema5a*), and receptors (e.g., *Mmd*, *Sstr2*) were consistently identified in *Foxg1*-deficient DG-GCs (Fig. 3C and fig. S4C).

It is worth mentioning that we also identified a set of subtype-specific genes with largely unknown functions, such as SubC-PyN–specific *B3gat1* (a glucuronyltransferase gene) and *Lix1* (a limb expression gene), CA1-PyN–specific *Rpp21* (a ribonuclease gene), CA3-PyN–specific *Spats2l* (a stress-responsive gene), and DG-GC–specific *Mmd* (a receptor) (Fig. 3C and fig. S4B). The CA3-PyN–specific gene *S100a10* (Fig. 3C and fig. S4B), a ligand involved in tumor progression (*37*), has been poorly studied in forebrain development. These subtype-specific genes may also contribute to the identities of distinct neuron subtypes. Collectively, we uncover subtype-specific FOXG1-driven transcriptional networks that define each distinct MP neuron subtype.

### Identification of cross-subtype FOXG1-driven candidate genes

A shared gene expression profile suggests that their activity is driven by the same factors and reflects their developmental and/or functional connections, and the expression levels of coexpressed genes across neuron subtypes need to be precisely controlled. To understand how gene-gene relationships shape MP neuron subtype identities, we next aimed to characterize FOXG1-driven subtype-coexpressed gene networks among 64 candidates. The TFs *Id2*, *Mef2c*, *Nme1*, and *Pou3f1*, which were enriched in both RSC-PyNs (UL) and SubC-PyNs, were severely down-regulated in all five subtype neurons in *Foxg1* cKO MPs (Fig. 3D and fig. S5A). *Bcl11b* and *Fezf2*, which were coexpressed in SubC-PyNs and CA1-PyNs, were down-regulated in both the two subtypes in *Foxg1* cKO MPs (Fig. 3D and fig. S5B). *Id2*, *Mef2c*, and *Nme1* have been shown to regulate synapse formation and neurite development (*38*–*40*). *Pou3f1* was reported to regulate transcription in differentiated neocortical neurons (*41*). *Bcl11b* and *Fezf2* are known to be the key determinants for cortical subcerebral projection neurons (*10*).

Cell adhesion genes, for instance, *Ntm* [coexpressed between RSC-PyNs (UL) and SubC-PyNs] was specifically down-regulated in these two subtypes in the absence of *Foxg1*, and *Cadm1* (enriched in both SubC-PyNs and CA3-PyNs) was down-regulated in all five subtype neurons after *Foxg1* deletion (Fig. 3D and fig. S5A). For cytoskeletal genes coexpressed between SubC-PyNs and CA3-PyNs, *Dlagap1*was up-regulated, whereas *Map1b* was down-regulated in all five subtype neurons in *Foxg1* cKO MPs (Fig. 3D and fig. S5C). Receptors, *Ptprz1*, and *Tenm3*, which were coexpressed between RSC-PyNs (UL) and SubC-PyNs, were specifically down-regulated in these two subtypes after *Foxg1* deletion (Fig. 3D and fig. S5A). A long noncoding RNA *Meg3* that showed high expression in both SubC-PyNs and CA3-PyNs was also identified to be up-regulated in all five subtype neurons after *Foxg1* deletion (Fig. 3D and fig. S5C). These dysregulated FOXG1 target genes may contribute to the abnormal identities of RSC-PyNs (UL), SubC-PyNs, CA1-PyNs, and CA3-PyNs in the *Foxg1* cKO MP.

Among TFs enriched in CA1-PyNs and CA3-PyNs, *Carhsp1*, *Zeb2*, and *Zbtb20* were up-regulated in all five subtype neurons in the *Foxg1* cKO MPs (Fig. 3D). *Zeb2* was previously reported to act upstream of Wnt signaling to control the formation of the hippocampus (*42*). However, the TF *Lmo1* was down-regulated in all five subtype neurons in *Foxg1* cKO MPs (Fig. 3D and fig. S6A). We also found that an ephrin receptor *Epha4*, previously shown to function in axon guidance and dendritic spine remodeling (*43*, *44*), was extensively up-regulated in all five subtype neurons in *Foxg1* cKO MPs (Fig. 3D). These data suggest that the expression levels of these subtype-coexpressed genes are precisely controlled by FOXG1 for the establishment of CA1-PyN and CA3-PyN identities.

Coexpressed genes in both CA3-PyNs and DG-GCs including TF *Neurod1*, *Nfib*, and *Prdm8*, and the receptor carboxypeptidase E (*45*), cell adhesion molecule *Nrxn1* (*46*), and potassium channel KCNK1 (*47*), which may contribute to the intrinsic excitability of DG-GCs, were up-regulated in all five subtype neurons after *Foxg1* deletion (Fig. 3D and fig. S6B). These up-regulated FOXG1 target genes may underline the severe expansion of the CA3-PyNs and DG-GCs in *Foxg1* cKO MPs.

Similar to the identification of subtype-specific genes with largely unknown functions, we also identified a set of dysregulated subtype-coexpressed candidate FOXG1 target genes with their functions remain unexplored, for instance, *Mpped1* (a metallophosphodiesterase gene) enriched in both RSC-PyNs (UL) and SubC-PyNs, *Rprm* (a tumor-suppressor gene) and *Ina* (a cytoskeletal gene) enriched in both SubC-PyNs and CA3-PyNs, and the TF *Carhsp1* showed high expression levels in both CA1-PyNs and CA3-PyNs. These genes may also orchestrate neuron subtype specification in the developing MP. Collectively, we reveal FOXG1-driven subtype-coexpressed gene networks that orchestrate the specification of MP neuron subtypes.

### Characterization of subtype-nonenriched FOXG1-driven gene networks

We next analyzed 228 subtype-nonenriched candidate FOXG1 target genes. We found that 132 genes, including 14 TFs, were up-regulated, while 96 genes, including 8 TFs, were down-regulated in *Foxg1* cKO MPs (Fig. 3E and table S3). Among up-regulated TFs, *Tbr1*, which is reported to regulate the fate of neocortical layer 6 projection neurons (*48*), was up-regulated in all five subtype neurons in the *Foxg1* cKO MP compared to limited expression in SubC-PyNs in the control MP (Fig. 3E and fig. S7A). *Cxxc5*, *Ier2*, and *Jarid2* were remarkably up-regulated in all five subtype neurons of *Foxg1* cKO MPs (Fig. 3E and fig. S7A). CXXC5 is reported to be involved in hippocampal development by acting as an inhibitor of Wnt signaling (*49*). Jumonji, AT rich interactive domain 2 (JARID2) is a regulator of histone methyltransferase complexes that inhibit transcriptional activity to allow the progression of embryonic cardiomyocyte differentiation (*50*). Among down-regulated TFs, *Bcl11a*, *Hmgcs1*, *Neurod6*, *Pbx1*, *Smarca2*, and *Sox4* were down-regulated in all five subtype neurons in *Foxg1* cKO MPs (Fig. 3E and fig. S7B). *Pou3f2*, which is expressed in RSC-PyNs, and *Pou3f3*, which is coexpressed in RSC-PyNs, SubC-PyNs and CA1-PyNs, were absent after *Foxg1* disruption (Fig. 3E and fig. S7B). These data suggest that FOXG1 orchestrates the expression of subtype-nonenriched FOXG1 target genes across MP neuron subtypes.

It is also worth mentioning that *Chd7*, *Dach1*, and *Ebf1*, which are usually expressed in MP progenitors, were substantially up-regulated in the entire *Foxg1* cKO MP (Fig. 3E and fig. S7A), which suggests that FOXG1 represses the progenitor molecular profile in postmitotic neurons. To examine whether *Foxg1* cKO postmitotic neurons acquired progenitor properties and to exclude the possibility that the impaired postmitotic subtype specification was caused by proliferation and neurogenesis defects, we performed immunostaining against KI67 and 5-bromo-2′-deoxyuridine (BrdU), and no notable differences were detected between the control and *Foxg1* cKO mice (fig. S8, A to C). Cell cycle exit did not change either when measured by the percentage of KI67^−^BrdU^+^ cells among total BrdU^+^ cells at E14.5 (fig. S8, D and E). We next performed a birth-dating experiment to trace neurogenesis and found no obvious changes (fig. S8, D and F). These results suggest that the proliferation and neurogenesis were not substantially affected after *Foxg1* deletion and that *Foxg1* cKO postmitotic neurons did not acquire progenitor properties. Together, we identified MP subtype-specific, subtype-coexpressed, and subtype-nonenriched candidate FOXG1 target genes that orchestrate the specification of neuron subtypes.

### Transcriptional repression at the *Zbtb20*, *Prox1*, and *Epha4* loci by FOXG1

**Fig. 4.**
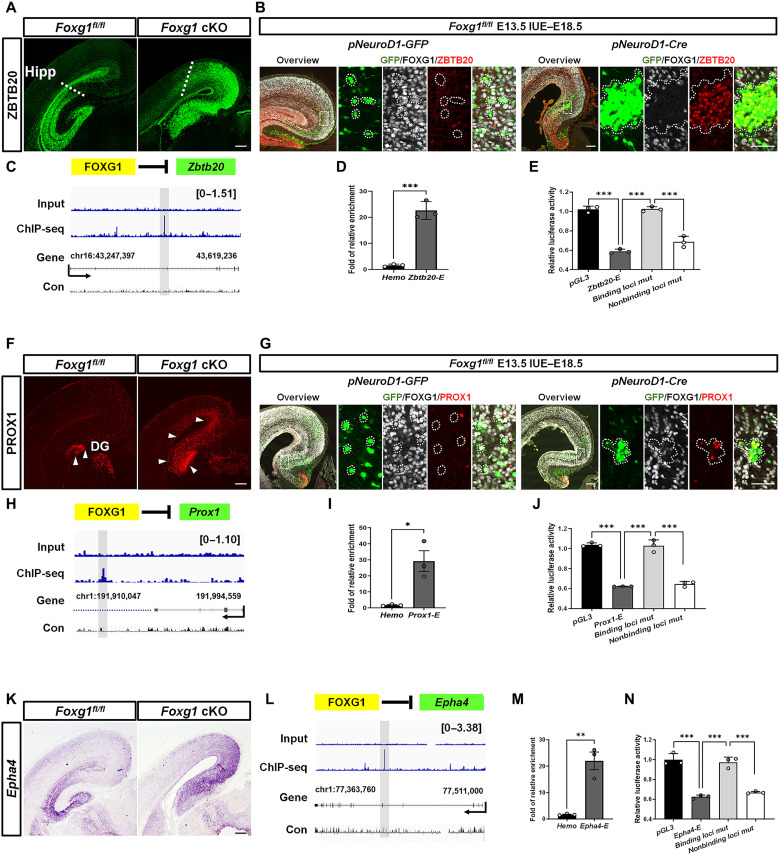
Transcriptional repression of FOXG1 at *Zbtb20*, *Prox1*, and *Epha4* loci. (**A**) Immunostaining at P0 showing ZBTB20 accumulation in the *Foxg1* cKO RSC and SubC. (**B**) Triple immunostaining at E18.5 showing efficient FOXG1 deletion and ZBTB20 accumulation in GFP^+^ neurons. (**C**) Genome browser view of FOXG1 ChIP-seq data at *Zbtb20* locus. (**D**) ChIP-qPCR showing high FOXG1 occupancy at *Zbtb20* locus. (**E**) Luciferase assay showing that FOXG1 directly represses *Zbtb20* and point mutations in the FOXG1-binding site weakens this repression. (**F**) Immunostaining at P0 showing PROX1 up-regulation in the entire *Foxg1* cKO MP. (**G**) Triple immunostaining at E18.5 after IUE, showing efficient deletion of FOXG1 and PROX1 accumulation in GFP^+^ neurons. (**H**) Genome browser view of FOXG1 ChIP-seq data at *Prox1* locus. (**I**) ChIP-qPCR showing high FOXG1 occupancy at *Prox1* locus. (**J**) Luciferase assay showing that FOXG1 directly represses *Prox1* and that point mutations at the FOXG1-binding site weaken this repression. (**K**) In situ hybridization at P0 showing *Epha4* up-regulation in the RSC and SubC of *Foxg1* cKO mice. (**L**) Genome browser view of FOXG1 ChIP-seq data at *Epha4* locus. (**M**) ChIP-qPCR showing high FOXG1 occupancy at *Epha4* locus. (**N**) Luciferase assay showing that FOXG1 directly represses *Epha4* and that point mutations in the FOXG1-binding site weaken this repression. Data are presented as the means ± SEM. (D, E, I, J, M, and N) Unpaired Student’s *t* test, *P < 0.05, **P < 0.01, and ***P < 0.001. Scale bars, 200 μm (A, B, F, G, and K) and 50 μm (B and G).

Among candidate DEGs, *Zbtb20*, *Prox1*, *Epha4*, *Nr4a2*, and *Tbr1* exhibit unique expression patterns in the developing MP and have been shown to be involved in subtype specification, making these genes potential downstream targets of FOXG1. *Zbtb20* expression is first detected in the hippocampal primordium as early as E12.5 and maintained to adulthood. In the absence of *Zbtb20*, the molecular border between the RSC, SubC, and hippocampal fields is progressively shifted ventrally with a reduced size of hippocampal fields (*6*), whereas ectopic expression of *Zbtb20* forces SubC-PyNs and RSC-PyNs (UL) into a hippocampus-like identity (*7*). Eph receptor A4 (EPHA4) is normally expressed throughout the entire developing hippocampus from early embryonic development. Previous studies have shown that EPHA4 functions in axon guidance and dendritic spine remodeling (*43*, *44*). NR4A2 is intensely expressed in the developing SubC and is reported to regulate SubC-PyN identity (*8*). T-box brain transcription factor 1 (TBR1) has been shown to be a master regulator of neocortical projection neurons (*48*). We then selected *Prox1* and *Nr4a2* from subtype-specific genes, *Zbtb20* and *Epha4* from subtype-coexpressed genes, and *Tbr1* from subtype-nonenriched genes for further assessments of FOXG1 transcriptional regulation. We first performed anti-ZBTB20 immunostaining at P0 and confirmed the substantially increased number of ZBTB20^+^ neurons in *Foxg1*-deficient MPs (Fig. 4A). Because *Foxg1* deletion also impairs neuron migration (*51*), to investigate whether FOXG1 cell-autonomously regulates *Zbtb20*, we then performed IUE to delete *Foxg1* at the RSC region locally. *pNeuroD1-Cre-GFP* DNA was delivered into the *Foxg1^fl/fl^* RSC at E13.5, and brains were examined at E18.5. We performed triple immunostaining against green fluorescent protein (GFP), FOXG1, and ZBTB20 and demonstrated that FOXG1 was efficiently deleted in *pNeuroD1-Cre-GFP*–transfected RSC-PyNs compared to its strong expression in the control MP (Fig. 4B). Notably, ZBTB20 was strongly up-regulated in *Foxg1-*deficient RSC-PyNs (Fig. 4B), demonstrating that ZBTB20 up-regulation was a cell-autonomous consequence of *Foxg1* deletion but not due to migration defects.

We then explored whether FOXG1 directly represses transcription from the *Zbtb20* locus. We conducted a bioinformatics analysis using the UCSC Genome Browser and identified apparent conservation of a putative FOXG1 motif binding site in the second intron of *Zbtb20* (Fig. 4C). ChIP-qPCR of E16.5 wild-type MPs with a FOXG1 antibody detected strong occupancy of FOXG1 at this site (Fig. 4D). A 24–base pair (bp) fragment containing the binding site for FOXG1 was cloned into a luciferase reporter plasmid and then transfected into N2A cells together with an expression plasmid for FOXG1. Luciferase assays showed that FOXG1 can directly suppress *Zbtb20* transcriptional activity. Point mutations in the binding site weakened the repression of FOXG1 on *Zbtb20* (Fig. 4E). These results support the notion that FOXG1 functions as a repressor of *Zbtb20*.

We next carried out immunostaining against PROX1 and confirmed its extensive expansion in the *Foxg1* cKO MP (Fig. 4F). Similarly, using IUE to delete *Foxg1* locally at the SubC area showed notable PROX1 up-regulation in *Foxg1*-deficient SubC-PyNs (Fig. 4G). We identified a putative consensus FOXG1-binding site within a distal 28-kb region of the *Prox1* locus (Fig. 4H). ChIP-qPCR revealed strong FOXG1 occupancy at this site at E16.5 (Fig. 4I). Furthermore, we cloned a 98-bp sequence containing a binding site for FOXG1 into the luciferase reporter plasmid and then transfected it into N2A cells together with the *Foxg1* expression plasmid. Luciferase assays revealed that FOXG1 functions as a direct repressor of *Prox1* transcription and that a point mutation in the FOXG1-binding site weakened this repression (Fig. 4J). These results demonstrate that FOXG1 directly represses *Prox1* transcription.

Similarly, in situ hybridization verified the remarkable expansion of *Epha4* to the SubC and RSC regions after *Foxg1* deletion (Fig. 4K). We identified a putative consensus FOXG1-binding site within the fourth intron of the *Epha4* locus (Fig. 4L). ChIP-qPCR detected a high enrichment of FOXG1 at the binding site (Fig. 4M). We cloned an 80-bp sequence containing a binding site for FOXG1 into the luciferase reporter plasmid and then transfected it into N2A cells together with the *Foxg1* expression plasmid. Luciferase assays revealed that FOXG1 represses *Epha4* transcriptional activity and that a point mutation in the FOXG1-binding site weakened this repression (Fig. 4N). Together, our results establish that FOXG1 transcriptionally represses *Zbtb20*, *Prox1*, and *Epha4* to prohibit the identities of CA3-PyNs and DG-GCs.

### Direct activation of *Nr4a2* by FOXG1 during the specification of SubC-PyNs

**Fig. 5.**
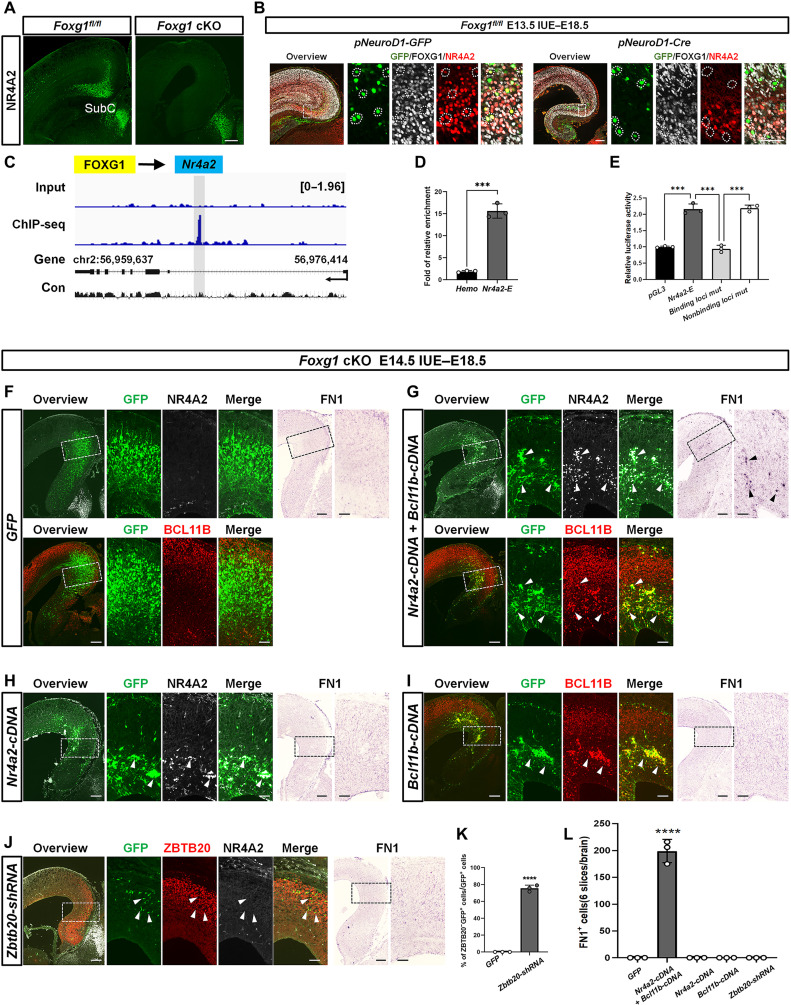
FOXG1 directly activates *Nr4a2.* (**A**) Immunostaining at P0 showing that NR4A2 level was remarkably reduced in *Foxg1* cKO SubC-PyNs. (**B**) Triple immunostaining at E18.5 after IUE showing the loss of NR4A2 in *Foxg1*-deficient GFP^+^ neurons. (**C**) Genome browser view of FOXG1 ChIP-seq data at *Nr4a2* locus. (**D**) ChIP-qPCR showing high FOXG1 occupancy at *Nr4a2* locus. (**E**) Luciferase assay showing that FOXG1 directly activates *Nr4a2* and point mutations in the FOXG1-binding site weaken this activation. (**F** and **G**) Immunostaining at E18.5 showing the restoration of NR4A2 and BCL11B after E14.5 IUE in *Foxg1* cKO mice. In situ hybridization showing *Fn1* expression is induced. (**H**) Immunostaining at E18.5 showing NR4A2 restoration alone after IUE in *Foxg1* cKO mice fail to induce *Fn1* expression. (**I**) Immunostaining at E18.5 showing BCL11B restoration alone after IUE in *Foxg1* cKO mice fail to induce *Fn1* expression. (**J**) Triple immunostaining at E18.5 showing that efficient *Zbtb20* knockdown after IUE in *Foxg1* cKO mice, no NR4A2 was detected. In situ hybridization showing that no *Fn1* was induced either. (**K**) Quantification analysis of the percentage of ZBTB20^−^GFP^+^ neurons among GFP^+^ cells showing that ZBTB20 was efficiently knocked down in *Zbtb20-shRNA–*transfected cells. (**L**) Quantification analysis of the number of *Fn1*^+^ neurons showing *Fn1* restoration. *n* = 3 brains and 6 slices per brain. Data are presented as the means ± SEM. (D, E, J, and K) Unpaired Student’s *t* test, ***P < 0.001 and ****P < 0.0001. Scale bars, 200 μm (A, B, F, G, H, I, and J) and 50 μm (B, F, G, H, I, and J).

To explore whether FOXG1 directly activates *Nr4a2*, we conducted immunostaining and confirmed the loss of NR4A2 expression in *Foxg1* cKO SubC-PyNs (Fig. 5A). Similarly, IUE demonstrated that the loss of NR4A2 in SubC-PyNs was a cell-autonomous consequence of *Foxg1* deletion but not due to migration defects (Fig. 5B). Using the UCSC Genome Browser, we identified an apparent conservation of the putative FOXG1-binding site in the first intron of *Nr4a2* (Fig. 5C). ChIP-qPCR with a FOXG1 antibody using E16.5 wild-type MP detected strong enrichment at the binding site (Fig. 5D). Luciferase assays further demonstrated that FOXG1 directly activated *Nr4a2*, and point mutations in binding motifs weakened the activating effect of FOXG1 on *Nr4a2* (Fig. 5E). These results demonstrate that FOXG1 directly activates *Nr4a2*.

It has been reported that forced expression of *Nr4a2* together with *Bcl11b* in RSC-PyNs induces SubC-PyN–specific *Fn1* expression (*8*). Recall that *Bcl11b* was substantially decreased in the *Foxg1* cKO MP (Fig. 3C and fig. S4B); we restored these two genes simultaneously into the *Foxg1* cKO SubC-PyNs by IUE. Strong *Fn1* expression was induced in SubC-PyNs, as shown by in situ hybridization (Fig. 5, F, G, and L). However, restoring either *Nr4a2* or *Bcl11b* alone failed to induce *Fn1* expression (Fig. 5, H, I, and L). These results suggest that FOXG1 and the downstream *Nr4a2 and Bcl11b* transcriptional networks are essential for the specification of SubC-PyN identity, and the cooperation of NR4A2 with BCL11B is required during this process.

A previous study showed that forced ectopic expression of *Zbtb20* results in the transformation of SubC-PyNs into a hippocampus-like identity (*7*), while loss of *Zbtb20* leads to NR4A2 up-regulation (*52*). To explore whether knockdown of *Zbtb20* in the *Foxg1* cKO MP could restore *Nr4a2* expression and could rescue SubC-PyN identity, *Zbtb20-shRNA* was electroporated into *Foxg1* cKO SubC-PyNs at E14.5 by IUE (Fig. 5I) after its knockdown efficiency was evaluated in cultured N2A cells (fig. S9A). Neither NR4A2 nor *Fn1* was detected in GFP^+^-*Zbtb20* knockdown cells (Fig. 5, J to L), indicating that ZBTB20 does not directly repress *Nr4a2* transcription. Our results suggest that FOXG1 may play a prominent role in regulating *Nr4a2* transcription during the specification of SubC-PyNs.

To further verify the activation of FOXG1 on *Nr4a2* and the repression on *Zbtb20*, *Prox1*, and *Epha4*, we quantified the fluorescence intensities of FOXG1 and its targets in NR4A2^+^ SubC-PyNs, PROX1^+^ DG-GCs, ZBTB20^+^ CA1-PyNs, CA3-PyNs, and DG-GCs. As expected, the expression level of FOXG1 was higher in NR4A2^+^ SubC-PyNs than that in PROX1^+^ DG-GCs (fig. S10, A to C). Notably, EPHA4 and ZBTB20 displayed a DG^high^-CA1^low^ gradient expression level when assessed by in situ hybridization and immunostaining, respectively, whereas FOXG1 exhibited a CA1^high^-DG^low^ complementary expression pattern (fig. S10, A, D, and E). We also carried out a gain of function of *Foxg1* by crossing *Nex-Cre* mice with a *CAG-loxp-stop-loxp-Foxg1-IRES-EGFP* line that we previously generated (*53*). We observed an obvious decrease in the expression levels of ZBTB20 and PROX1 as well as a slightly increased NR4A2 expression levels in the *Nex-Cre; CAG-loxp-stop-loxp-Foxg1-IRES-EGFP* MP (fig. S11A). Western blotting at P0 further confirmed the down-regulation of ZBTB20 and PROX1 and the up-regulation of NR4A2 after *Foxg1* overexpression (fig. S11B). These results collectively support the activation of FOXG1 on *Nr4a2* and the repression on *Zbtb20*, *Prox1*, and *Epha4*.

### Down-regulation of *Tbr1* during the specification of CA1-PyN

**Fig. 6.**
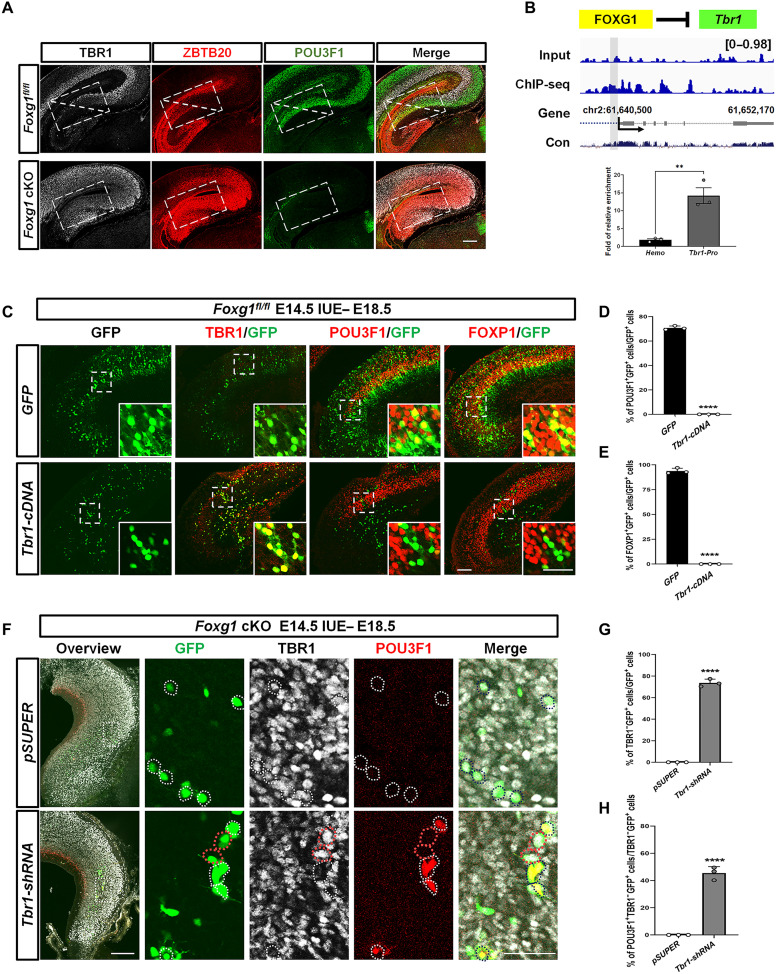
TBR1 should be down-regulated during the specification of CA1-PyNs. (**A**) Triple immunostaining against TBR1, ZBTB20, and POU3F1 at P0 showed the complementary expression pattern of TBR1 and ZBTB20 without overlapping and the strong expression of POU3F1 in CA1-PyNs in the control mice, whereas in *Foxg1* cKO MP, TBR1 was colocalized with ZBTB20, both TBR1 and ZBTB20 were substantially up-regulated, and POU3F1 was completely lost. (**B**) Genome browser view of FOXG1 ChIP-seq data for the binding site at 700-bp upstream of the *Tbr1* transcription start site. ChIP-qPCR with E16.5 MP samples showing high FOXG1 occupancy at the promotor of *Tbr1*. (**C**) Immunostaining against GFP, TBR1, POU3F1, and FOXP1 at E18.5 showing successful misexpression of TBR1 in CA1-PyNs and the down-regulation of POU3F1 and FOXP1 in *Tbr1-*misexpressing GFP^+^ cells. (**D**) Quantification analysis of the percentage of POU3F1^+^GFP^+^ neurons among GFP^+^ neurons at E18.5 showing that no POU3F1 was detectable in *Tbr1-cDNA*–transfected cells. (**E**) Quantification analysis of the percentage of FOXP1^+^GFP^+^ neurons among GFP^+^ neurons at E18.5 showing that no FOXP1 was detectable in *Tbr1-cDNA*–transfected cells. (**F**) Triple immunostaining against GFP, TBR1, and POU3F1 in MP at E18.5 showing POU3F1 up-regulation in *Tbr1* knockdown neurons by IUE in *Foxg1* cKO mice. (**G**) Quantification analysis of the percentage of TBR1^−^GFP^+^ neurons among GFP^+^ neurons showing that *Tbr1* was knocked down in approximately 80% of total transfected cells. (**H**) Quantification analysis of the percentage of POU3F1^+^TBR1^−^GFP^+^ neurons among TBR1^−^GFP^+^ neurons showing that total *Tbr1* knockdown POU3F1 cells were restored in 45% of cells. Data were presented as the means ± SEM. (D, E, G, and H) Unpaired Student’s *t* test, **P < 0.01, ****P < 0.0001. Scale bars, 200 μm (in overview of A, C, and F) and 50 μm (the magnification of C and F).

Among identified DEGs, *Tbr1* was severely up-regulated in *Foxg1* cKO mice (Fig. 3E and fig. S7A). Previous studies have shown that TBR1 regulates the regional and laminar identity of postmitotic neurons in the neocortex (*48*). In the control, developing MP TBR1 was expressed in RSC-PyNs and SubC-PyNs but not the CA region or the DG. However, we detected severe expansion of TBR1 to the entire *Foxg1* cKO MP at P0 by immunostaining (Fig. 6A). ZBTB20 is reported to define a hippocampal identity through direct repression of *Tbr1* (*54*). Notably, the two TFs, TBR1 and ZBTB20, exhibited a complementary expression pattern without overlap in the control mice; however, TBR1 displayed abnormal colocalization with ZBTB20 in the *Foxg1* cKO mice (Fig. 6A). It has been shown that deletion of *Tbr1* leads to up-regulation of *Pou3f1* (*55*), a commonly used marker for CA1-PyNs. At P0, immunostaining showed that the specific expression of POU3F1 was absent in the *Foxg1* cKO MP (Fig. 6A). These observations suggest that for the establishment of CA1-PyN identity, TBR1 needs to be down-regulated.

To explore whether FOXG1 directly represses *Tbr1* during the specification of CA1-PyNs, we first performed ChIP-qPCR and detected a strong FOXG1 occupancy at a putative binding site 700 bp upstream of the *Tbr1* transcription start site, as previously reported (*10*) (Fig. 6B). We then misexpressed *Tbr1* in the control CA1-PyNs by IUE at E14.5 to mimic TBR1 up-regulation in the *Foxg1* cKO MP. As expected, both POU3F1 and FOXP1 (another marker for CA1-PyNs) were lost in cells in which *Tbr1* was misexpressed, as viewed by immunostaining at E18.5 (Fig. 6, C to E), suggesting that TBR1 prohibits CA1-PyN identity. Given that we previously demonstrated that FOXG1 directly represses *Tbr1*, it is possible that establishing CA1-PyN identity requires the repression of FOXG1 on *Tbr1*. We next delivered *Tbr1-shRNA* to knock down *Tbr1* in *Foxg1* cKO CA1-PyNs by IUE at E14.5. When brains were examined at E18.5, we found that POU3F1 was restored in 45% of *Tbr1* knockdown CA1-PyNs (Fig. 6, F to H, and fig. S7B). Collectively, these results demonstrate that the establishment of CA1-PyN identity requires TBR1 down-regulation, which is controlled by FOXG1. Altogether, we demonstrate that FOXG1 specifies MP principal neuron subtypes by controlling subtype-specific, subtype-coexpressed, and subtype-nonenriched transcriptional programs (fig. S12).

## DISCUSSION

To date, great progress has been made in the establishment of neuronal diversity in the neocortex and the ventral pallium; however, few studies have examined the specification of MP principal neuron subtypes. Here, we show evidence that FOXG1 orchestrates the postmitotic specification of MP neuron subtypes. We uncovered FOXG1-driven transcriptomic networks and identified genes whose roles have not yet been examined in developing MP. The present data therefore expand our understanding of the mechanism underlying MP development.

In the present study, we identified a set of previously unidentified candidate FOXG1 target TFs that are specifically expressed in distinct MP neuron subtypes and showed severe changes after *Foxg1* deletion. For example, *Myt1l*, *Lmo4*, and *Zfp462* in RSC-PyNs (UL); *Ssbp2* in SubC-PyN; *Lmo7*, *Insm1*, and *Foxp1* in CA1-PyNs; and *Zfpm2*, *Nhlh2*, and *Hmgb3* in DG-GCs. Future studies exploring the roles of these TFs in distinct neuron subtypes would provide more insights into the TF regulatory network controlling MP development. We also identified a set of genes, such as *Chd7*, *Ebf1*, and *Dach1*, which are usually expressed in MP progenitors and were remarkably up-regulated in postmitotic neurons in the *Foxg1* cKO MP, suggesting a prominent suppression of FOXG1 on progenitor properties to specify the postmitotic neurons. Our findings thus provide important molecular clues for future studies on MP development.

In this study, scRNA-seq was performed at E17.5, a time point at which the majority of MP principal neurons have already been born and undergone postmitotic specification (*14*). Candidate genes identified at this developmental stage would represent the most important transcriptomic networks orchestrating MP neuron specification regulated by FOXG1. In the absence of FOXG1, many target genes—such as *Zbtb20*, *Prox1*, *Epha4*, *Nr4a2*, *Tbr1*, and *Satb2*—were consistently up-regulated/down-regulated from the embryonic to postnatal stages, suggesting their prominent roles in the postmitotic subtype specification. However, given that the expression of genes during neural development is highly dynamic and occurs within a fine spatial and temporal regulation framework, whether some DEGs identified at E17.5 that remain differentially expressed at other time points remains to be explored in the future.

Previous studies have shown that SATB2 is required for the specification of RSC neurons. RSC-PyNs (UL) are absent in *Satb2* mutant mice (*8*). Here, we showed a loss of RSC-PyNs (UL) and marked down-regulation of SATB2 in *Foxg1* cKO mice. When viewed alongside our earlier findings that FOXG1 specifies neocortical callosal projection neurons through direct activation of *Satb2* (*10*), our data support the notion that FOXG1 may also specify RSC-PyNs (UL) by acting as a transcriptional activator of *Satb2*.

Although SubC, CA1, CA3, and the DG are all components of the archicortex, our results reveal that FOXG1 prohibits the identities of CA3-PyNs and DG-GCs but promotes SubC-PyNs and CA1-PyNs. FOXG1 also promotes the identity of intermediate cortical RSC-PyNs (UL). Because SubC functionally constitutes a relay point between the hippocampus CA1 (archicortex) and the RSC (intermediate cortex) and the coexpressed genes in SubC-PyNs with RSC-PyNs (UL) and CA1-PyNs reflect closely linked developmental and evolutionary interconnections among these principal neuron subtypes, it seems likely that further exploration of the roles of these coexpressed genes could yield insights into the developmental and evolutionary correlations of the circuit of CA1-PyNs–SubC-PyNs–RSC-PyNs (UL).

CA1-PyNs and CA3-PyNs share higher gene expression similarities than other principal neuron subtypes in the MP, which is in line with their similar morphology, function, and regional localization. It is quite informative that in the absence of *Foxg1*, the majority of genes specific to CA1-PyNs were severely down-regulated, whereas genes specific to CA3-PyNs were massively up-regulated. Among the eight genes coexpressed between the two subtypes, four were up-regulated, whereas four were down-regulated. It should also be noted that the earliest CA1-PyN– and CA3-PyN–specific markers appear first at the poles of the hippocampus and move inward (*56*). Future studies using cell type–specific loss-of-function strategies would address how these subtype-specific genes and subtype-coexpressed genes shape CA1-PyNs and CA3-PyNs, respectively.

Previously, we showed severe up-regulation of *Bcl11b* in the neocortex after postmitotic deletion of *Foxg1* using the same *Nex-Cre* line used in the present study and demonstrated that FOXG1 directly represses *Bcl11b* by binding to its distal repressor site during specification of neocortical neurons (*10*). Although we consistently detected severe up-regulation of BCL11B in the neocortex in the present study, intriguingly, BCL11B was substantially decreased in the *Foxg1* cKO MP. This observation suggests that FOXG1 may drive two different mechanisms to shape the neocortical and MP principal neuron subtypes, and there are some as-yet-unknown partners cooperating with FOXG1 to precisely regulate *Bcl11b* transcription during the specification of MP principal neuron subtypes. Future works using liquid chromatography–tandem mass spectrometry to characterize different partners would be particularly interesting and could help to elucidate the mechanisms underlying neocortical and MP neuron subtype specification.

ZBTB20 is normally expressed in the hippocampus in a DG^high^-CA1^low^ manner. Previous studies have shown that ZBTB20 is required for the establishment of archicortical arealization. Deletion of *Zbtb20* causes a progressive ventral displacement and molecular misspecification of all MP domains, and hippocampal fields including CA1, CA3, and DG are severely reduced in size (*6*). In the present study, despite the notable up-regulation of ZBTB20 in the *Foxg1* cKO MP, the majority of genes specific to CA1-PyNs were down-regulated. In contrast, genes specific to CA3-PyN were substantially up-regulated. It seems that during the specification of CA1-PyNs and CA3-PyNs, ZBTB20 may promote genes coexpressed between CA1-PyNs and CA3-PyNs, whereas activation of genes specific to CA1-PyNs may require FOXG1. It should also be noted that *Zbtb20* expression extensively spreads to the SubC and RSC regions, and SubC-PyNs and RSC-PyNs were lost in the *Foxg1* cKO MP. Our results are consistent with previous studies showing that forced ectopic expression of *Zbtb20* enforces SubC-PyNs into a hippocampus-like identity (*7*).

EPHA4 was selectively expressed in the hippocampus. EPHA4 plays an important role in axon guidance and synaptic plasticity. In *Epha4* KO mice, the dendritic spines of CA1-PyNs and CA3-PyNs were irregular in shape and exhibited a disorganized appearance (*43*), and mossy fiber was much thinner and less robust than that of the control mice (*44*). In the present study, EPHA4 was substantially up-regulated in *Foxg1* cKO MPs, which may contribute to the abnormal development of projections of MP principal neurons. PROX1 is specifically expressed in DG-GCs and is a key cell fate determinant for DG-GCs. Postmitotic *Prox1* KO deprives the DG-GC signature of gene expression. Ectopic *Prox1* expression favors DG-GC differentiation over the pyramidal cell fate (*5*). In *Foxg1* cKO MPs, *Prox1* expression expanded into the deep SubC and RSC regions, coincident with the increased DG-GCs. Our data provide evidence that FOXG1 functions upstream of *Prox1* to specify DG-GCs.

Cytoarchitectonic changes in the hippocampus, SubC and RSC, were reported in a postmortem study of autism spectrum disorders (*57*). Dysfunctions in the hippocampus, SubC or RSC, have also been repeatedly found in patients with schizophrenia and epilepsy (*58*). *FOXG1* has been implicated in a wide spectrum of congenital brain disorders, including autism spectrum disorder, schizophrenia, and epilepsy (*59*). We uncover a set of genes that are identified to be closely linked to developmental disorders. For instance, *Tbr1*, *Chd7*, and *Hdac2* are autism spectrum disorder risk genes (*48*, *60*, *61*). *Nrxn1* and *Meg3* are specifically linked to schizophrenia (*46*, *62*). *Zeb2*, *Pcdh19*, and *Bcl11a* are epilepsy-associated genes (*63*–*65*). Thus, our study sheds light on the pathogenesis of MP-associated high brain function deficits and related neurodevelopmental disorders.

## MATERIALS AND METHODS

### Animals

*Foxg1^fl/fl^* mice and *CAG-loxp-stop-loxp-Foxg1-IRES-EGFP* mice were generated as previously described (*53*). *Nex-Cre* mice (stock: 2668659) were purchased from The Jackson Laboratory. *Nex-Cre; Foxg1^fl/fl^* mice were referred to as *Foxg1* cKO, and *Foxg1^fl/fl^* mice were referred to as control. The day of the vaginal plug detection was assigned as E0.5, and the day of birth was assigned as P0. Unless noted otherwise, all experiments were performed using mice maintained on a CD1 background. No notable differences based on sex were observed, and data were pooled between sexes. All mouse studies were approved by the Southeast University Institutional Animal Care and Use Committee and were performed in accordance with institutional and national guidelines.

### Immunofluorescence and in situ hybridization analyses

Mice at E16.5, P0, and P4 were perfused with 4% paraformaldehyde (PFA), and brains were then dissected out, postfixed overnight, cryoprotected in 30% sucrose, and sectioned on a cryostat (Leica, CM 3050S). Immunofluorescence staining was performed as previously described (*10*): Briefly, sections were permeabilized in phosphate-buffered saline (PBS) containing 0.1% Triton X-100 for 0.5 hours, blocked with 10% calf serum for 2 hours at room temperature (RT), and incubated in primary antibody diluted in blocking solution overnight at 4°C. Sections were then incubated in secondary antibody for 2 hours at RT. Images were captured with a confocal microscope (FLUOVIEW FV1000, Olympus). Antibodies for immunofluorescence were listed in table S4.

For in situ hybridization, simply, probes were obtained by PCR amplification, and primers for PCR amplification are listed in table S5. Brain sections were hybridized with digoxigenin-labeled antisense RNA probes at 65°C overnight, incubated with anti–digoxigenin–alkaline phosphatase antibody for 2 hours at RT, and then subjected to color development.

### Cell tracing and BrdU immunofluorescence

For BrdU (Sigma-Aldrich, catalog no. B5002) labeling, timed pregnant females were intraperitoneally administered with a dose of 50 mg/kg bodyweight at E14.5. Brains of paired littermates were examined at 0.5 and 24 hours after injection or at P0. As for BrdU immunofluorescence, sections were fixed in 4% PFA for 20 min at RT after aforementioned incubation, treated with 2 N HCl at 37°C for 0.5 hours, rinsed three times in PBS, and then incubated with anti-BrdU antibody overnight at 4°C followed by secondary antibody incubation for 2 hours at RT.

### Nissl staining

Nissl staining was performed as previously described (*66*). Brain slices were first immersed in distilled water for 2 min, transferred to Nissl staining solution for 30 min, and immersed in 95% alcohol for 5 s. The slices were then dipped in xylene for 5 min and sealed with rhamsan gum.

### Western blotting analyses

Western blotting was performed according to a standard protocol. MP extracts at P0 were separated on 10% SDS–polyacrylamide gel electrophoresis gel and transferred to polyvinylidene fluoride membranes. The membranes were blocked for 2 hours in 5% nonfat dry milk containing 20 mM tris-HCl (pH 7.5), 150 mM NaCl, and 0.1% Tween 20 and incubated with primary antibodies for overnight at 4°C followed by secondary horseradish peroxidase–linked anti-rabbit immunoglobulin G (IgG) incubation for 2 hours at RT. Proteins were detected using the Chemiluminescent SuperSignal West Dura Kit (Thermo Fisher Scientific).

### Tissue processing for scRNA-seq

E17.5 brains from *Foxg1^fl/fl^* (control) and *Nex-Cre; Foxg1* cKO mice were dissected out and immediately submerged in fresh ice-cold Hanks’ balanced salt solution (Gibco, 12175-095). MPs were then cut into pieces and dissociated into a single-cell suspension using the Papain Cell Dissociation Kit (Miltenyi Biotec, catalog no. 130-092-628) by following the manufacturer’s instruction. The Chromium droplet-based sequencing platform (10X Genomics) was used to generate scRNA-seq libraries, following the manufacturer’s instructions (manual document part number: CG00052 Rev C). The cDNA libraries were purified, quantified using an Agilent 2100 Bioanalyzer, and sequenced on an Illumina HiSeq 4000.

### scRNA-seq analysis

High-quality sequences (clean reads) of two samples, *Foxg1^fl/fl^* (control) and *Nex-Cre; Foxg1* cKO (exp), were processed by Cell Ranger to obtain quantitative information of gene expression. Cell quality control thresholds were set to 750 to 5000 genes per cells and <10% of mitochondrial transcripts. A global-scaling normalization method “log normalize” was applied to the raw read counts. Find Integration Anchors and Integrate Data function were then used to combine the two normalized datasets. Statistically significant principal components determined by a resampling test were kept for UMAP analysis. The Wilcoxon rank sum test was used to identify enriched genes (adjusted *P* value < 0.05, |log_2_FC| > 0.25) in MP principal neuron subtype clusters. For cluster annotation, the most comprehensive and reliable cell type marker was sought through extensive literature review. Within DEG analysis (adjusted *P* value < 0.05, |log_2_FC| > 0.3) between genotypes, neurons identified as either PyNs or GCs regardless of cluster were combined into pools of cells (*67*). All these analyses were performed in the Seurat package v4.0.6 (https://satijalab.org/seurat/).

### Functional annotation GO term analysis

ClusterProfiler, GO.db, DOSE, org Mm.eg.db, and org.Hs.eg.db packages in R were used for functional annotation of the list of expressed genes, and Benjamini-Hochberg algorithm was used to control *P* value adjustment. After the adjustment, GO item with *P* value < 0.05 was judged as significant item.

### Annotation of genes using existing datasets

Integration of TF annotations with three databases, TRRUST2, TFdb, and GO. TRRUST2, (www.grnpedia.org/trrust/) is a manual management of human and mouse across the regulation network of text database. TFdb (http://gerg.gsc.riken.jp/TFdb/) is a database of mouse TF genes and related genes. These genes are labeled GO:0003700, “DNA binding TF activity”, in the GO database.

### Identification of FOXG1-binding sites

Binding sites of FOXG1 at *Zbtb20*, *Prox1*, *Epha4*, and *Nr4a2* loci were identified by publicly available FOXG1 ChIP-seq data (GEO database, GSE96070) (*16*), and the conservation of the consensus binding sites of RYAAAYA of FOXG1 at each putative regulatory locus was further evaluated across species at the UCSC database (http://genome.ucsc.edu/) as previously reported (*10*).

### ChIP-qPCR assay

ChIP was carried as described previously with some modification (*10*). Briefly, E16.5 wild-type mouse MPs were mechanically homogenized, and samples were cross-linked with 1% formaldehyde for 10 min and quenched with 2.5 M glycine for 5 min. The cross-linked chromatin was sonicated to generate fragments with an average length of 300 to 400 bp. The chromatin was subjected to immunoprecipitation using 3 μg of anti-FOXG1 antibodies or anti-IgG antibodies. After reversal of cross-links, DNA and 10% input samples were purified using a QIA quick PCR purification kit (QIAGEN, 28106). For quantitative reverse transcription PCR analyses, primers (listed in table S5) were designed within 300 to 500 bp of the FOXG1-binding sites, the *Hemo* gene served as a negative control for ChIP-qPCR. All experiments were run in duplicate and repeated three independent times.

### Luciferase assay

The luciferase assay was performed as previously described (*10*). The overexpression vector of *pCAG-Foxg1* (mouse, NM_008241) was customized from SYNBIO TECH (www.synbio-tech.com; Beijing, China). Reporter vectors of *pGL3-Zbtb20-Pro* (chr16:43,525,750-43,525,873), *pGL3-Prox1-Pro* (chr1:191,994,541-191,994,626), *pGL3-Epha4-Pro* (chr1:77,423,646-77,423,725), and *pGL3-Nr4a2-Pro* (chr2:56,973,221-56,973,316) were homemade, in which fragments containing FOXG1-binding site were cloned into the *pGL3-Promoter* vector (primers listed in table S5). The point-mutated reporter constructs were generated using the Mut Express II Fast Mutagenesis Kit V2 (Vazyme, catalog no. C214-01). Mutation information was presented in table S6. All of the constructs were verified by sequencing.

Neuro2a cells (RRID: CVCL0470, the cell bank of the Chinese Academy of Science, Shanghai, China) were cultured in Dulbecco’s modified Eagle’s medium/F12 (Gibco, catalog no. 11320082) containing 10% fetal bovine serum (Gibco, catalog no. 10099141C), penicillin (100 U/ml), and streptomycin (100 μg/ml) (Millipore, catalog no. 1516104-M). A total of 1 × 10^5^ cells were seeded for transfection. The transfection complex was prepared including *pCAG* vector (control) or overexpression vector *pCAG-Foxg1*(300 ng per cell), reporter vectors or mutation reporter vector (300 ng per cell), *pRL-SV40* plasmid containing Renilla luciferase gene (30 ng per cell), and Lipofectamine 2000 (2.5 μl per cell) (Thermo Fisher Scientific, catalog no. 11668-019). For normalization of luciferase activity, the *pRL-SV40* plasmid encoding Renilla luciferase was used as an internal control reporter and was used to combination with any experimental reporter vector to cotransfect mammalian cells. Cells were harvested 24 hours after transfection. Firefly and Renilla luciferase activities were measured using a Dual Luciferase Reporter Assay System (Promega, E1910). The ratio of firefly luciferase readouts and Renilla luciferase readout was calculated to represent the activity of reporter vector. Three replicate wells were made for each transfection. Each experiment was repeated at least three times, and all results are shown as the means ± SEM.

### RNA isolation, reverse transcription, and qPCR analysis

RNA isolation, reverse transcription, and qPCR were performed as previously described (*10*). Neuro2a cells were harvested 72 hours after *Zbtb20-shRNA* or *Tbr1-shRNA* transfection. Total RNA was extracted using the RNeasy Plus Mini Kit (QIAGEN, 74104) and was reverse-transcribed using the PrimeScript RT reagent Kit with gDNA Eraser (Takara, RR047A). qPCR reactions were performed using SYBR Green fluorescent master mix (Roche, catalog no. 04707516001) on a Step One-Plus Real-Time System (Applied Biosystems). Relative mRNA expression levels were normalized with the expression of glyceraldehyde-3-phosphate dehydrogenase. The primers for qPCR were listed in table S5. Three replicate wells were made for each transfection, and samples were run in three duplicates. All of *P* values were presented as means ± SEM.

### In utero electroporation

To disrupt *Foxg1* in RSC-PyNs or SubC-PyNs locally, pregnant *Foxg1^fl/fl^* female mice (E13.5) were deeply anesthetized by 0.8% pelltobarbitalum natricum (Merck, TF110), and embryos were surgically manipulated as described previously (*10*). *pNeuroD1-Cre-HA-IRES-GFP* plasmid (*pNeuroD1-Cre*) and control plasmid *pNeuroD1-IRES-GFP* (*pNeuroD1-GFP*) (2 μg/μl) were injected directly into the lateral ventricles of the *Foxg1^fl/fl^* embryonic brains. To restore NR4A2/BCL11B or knock down *Zbtb20* in *Foxg1* cKO SubC-PyNs, plasmids of *pCAG-Nr4a2*-cDNA (1 μg/μl) or/and *pCAG-Bcl11b*-cDNA (1 μg/μl) or *Zbtb20-shRNA* (2 μg/μl) were injected directly into the lateral ventricles of E14.5 *Foxg1* cKO embryos. *pCAG-GFP* (1 μg/μl) was used as the control reporter vector. For ectopic expressing TBR1 in CA1-PyNs, plasmid *pCAG-Tbr1-cDNA* (1 μg/μl) or control *pCAG-GFP* was injected into the lateral ventricles of the wild-type embryonic brains at E14.5. For knocking down *Tbr1* at E14.5 in *Foxg1* cKO CA1-PyNs, control *pSURPER* (2 μg/μl) or *Tbr1-shRNA* plasmids (2 μg/μl) were injected.

For construction of IUE plasmids, *Zbtb20*-shRNA was targeted against 5′-GCAAAGGGTGCAGATCCTAGA-3′ (nucleotides 1561 to 1583 of NM_019778); *Tbr1-shRNA* was targeted against 5′-GGAGGCAAATGGGTTCCTTGT-3′ (nucleotides 1057 to 1078 of NM_009322.3). The complementary oligonucleotides were annealed and inserted into the *pSUPER-EGFP* vector (*pSUPER* for short), which itself alone was used as the control. *pCAGIG-Tbr1-cDNA* plasmid (*Tbr1-cDNA*, NM_009322) was customized from the You Bio Company (www.youbio.cn/; ChangSha, China). Plasmids of *pCAG-Nr4a2-cDNA* (NM_013613) and *pCAG-Bcl11b-cDNA* (NM_001079883) were gifts from Y.-Q. Ding at Fudan University.

Electroporation was performed using Tweezertrodes (diameter, 5 mm; BTX, Holliston, MA, USA) with five pulses (34 V for E13.5 embryos and 36 V for E14.5 embryos) for 50-ms duration and 950-ms intervals using a square wave pulse generator (ECM830, BTX). The uterus was then returned to the abdominal cavity, and the inner skin and outer skin were sutured. The surgically manipulated pregnant mice were put on an electric heating plate (50°C) until waking up. Progenies were euthanized at E18.5 for analysis. Three animals for each genotype were used.

### Statistical analyses

The confocal images for quantitative analyses were acquired using FLUOVIEW FV1000 confocal microscopy (Olympus) with a 20× objective lens. A minimum of six successive coronal sections crossing the MP were selected. Quantitative analyses of neurons were consistently performed in the same area in the MP by direct comparisons of brain sections. At least three brains of each genotype were used. The experiments of cell counting all were cross-quantified blindly (i.e., the investigator was unaware of which of the sections and areas were collected from). All results are shown as means ± SEM, except indicated otherwise. GraphPad Prism 7 software was used for statistical analyses, and the unpaired Student’s *t* test was used for two group comparisons. The following convention was used: *P < 0.05; **P < 0.01; ***P < 0.001; ****P < 0.0001; and ns, not significant.

The data of ChIP-qPCR and luciferase assay were analyzed with Student’s *t* test for two-group comparisons (Figs. 4, D, E, I, J, M, and N, and 5, D and E).

For quantification of electroporated cell number in MPs, all ZBTB20^−^GFP^+^ cells, GFP^+^ cells, *Fn1*^+^ cells, POU3F1^+^GFP^+^ cells, FOXP1^+^GFP^+^ cells, TBR1^−^GFP^+^ cells, and POU3F1^+^TBR1^−^GFP^+^ cells were counted, and respective ratios were calculated (Figs. 5, J and K, and 6, D, E, G, and H).

For quantitation of the proliferation number and differentiation ratios of MP progenitors, cells were counted in a radial column within a 200-μm-wide area in MP each section. The number of KI67^+^ cells, BrdU^+^ cells, KI67^−^BrdU^+^ cells, and TUJ1^+^BrdU^+^ cells was counted, and respective ratios were calculated (fig.S8, B, C, E, and F).

For quantification of fluorescence intensity, sections were imaged with the same confocal acquisition parameters at the same level for each of the individual protein assessed with immunostaining. The mean fluorescence intensities were analyzed using FV10-ASW4.2 software, with the same settings. The mean fluorescence intensities of FOXG1 and NR4A2 in SubC-PyNs, FOXG1 and PROX1 in DG-GCs, FOXG1 and ZBTB20 in CA1-PyNs, CA3-PyNs, and DG-GCs at P0 were measured, respectively. At least 500 neurons from three control brains for each population were analyzed (fig. S10, B to E).

## Notes

View/request a protocol for this paper from *Bio-protocol*.

## References

[1] N. Matsumoto, T. Kitanishi, K. Mizuseki, The subiculum: Unique hippocampal hub and more. Neurosci. Res. 143, 1–12 (2019). 30121285 10.1016/j.neures.2018.08.002

[2] S. D. Vann, J. P. Aggleton, E. A. Maguire, What does the retrosplenial cortex do? Nat. Rev. Neurosci. 10, 792–802 (2009). 19812579 10.1038/nrn2733

[3] A. J. Nelson, A. L. Powell, J. D. Holmes, S. D. Vann, J. P. Aggleton, What does spatial alternation tell us about retrosplenial cortex function? Front. Behav. Neurosci. 9, 126 (2015). 26042009 10.3389/fnbeh.2015.00126 PMC4435072

[4] J. B. Angevine Jr., Time of neuron origin in the hippocampal region. An autoradiographic study in the mouse. Exp. Neurol. Suppl. Suppl 2, 1–70 (1965). 5838955

[5] T. Iwano, A. Masuda, H. Kiyonari, H. Enomoto, F. Matsuzaki, Prox1 postmitotically defines dentate gyrus cells by specifying granule cell identity over CA3 pyramidal cell fate in the hippocampus. Development 139, 3051–3062 (2012). 22791897 10.1242/dev.080002

[6] E. H. Rosenthal, A. B. Tonchev, A. Stoykova, K. Chowdhury, Regulation of archicortical arealization by the transcription factor Zbtb20. Hippocampus 22, 2144–2156 (2012). 22689450 10.1002/hipo.22035

[7] J. V. Nielsen, F. H. Nielsen, R. Ismail, J. Noraberg, N. A. Jensen, Hippocampus-like corticoneurogenesis induced by two isoforms of the BTB-zinc finger gene Zbtb20 in mice. Development 134, 1133–1140 (2007). 17301088 10.1242/dev.000265

[8] L. Zhang, N.-N. Song, Q. Zhang, W.-Y. Mei, C.-H. He, P. Ma, Y. Huang, J.-Y. Chen, B. Mao, B. Lang, Y.-Q. Ding, Satb2 is required for the regionalization of retrosplenial cortex. Cell Death Differ. 27, 1604–1617 (2020). 31666685 10.1038/s41418-019-0443-1 PMC7206047

[9] B. Martynoga, H. Morrison, D. J. Price, J. O. Mason, Foxg1 is required for specification of ventral telencephalon and region-specific regulation of dorsal telencephalic precursor proliferation and apoptosis. Dev. Biol. 283, 113–127 (2005). 15893304 10.1016/j.ydbio.2005.04.005

[10] J. Liu, M. Yang, M. Su, B. Liu, K. Zhou, C. Sun, R. Ba, B. Yu, B. Zhang, Z. Zhang, W. Fan, K. Wang, M. Zhong, J. Han, C. Zhao, FOXG1 sequentially orchestrates subtype specification of postmitotic cortical projection neurons. Sci. Adv. 8, eabh3568 (2022). 35613274 10.1126/sciadv.abh3568 PMC9132448

[11] S. Goebbels, I. Bormuth, U. Bode, O. Hermanson, M. H. Schwab, K.-A. Nave, Genetic targeting of principal neurons in neocortex and hippocampus of NEX-Cre mice. Genesis 44, 611–621 (2006). 17146780 10.1002/dvg.20256

[12] C.-M. Chen, H.-Y. Wang, L.-R. You, R.-L. Shang, F.-C. Liu, Expression analysis of an evolutionarily conserved metallophosphodiesterase gene, Mpped1, in the normal and beta-catenin-deficient malformed dorsal telencephalon. Dev. Dyn. 239, 1797–1806 (2010). 20503375 10.1002/dvdy.22293

[13] K. Bermudez-Hernandez, Y.-L. Lu, J. Moretto, S. Jain, J. J. LaFrancois, A. M. Duffy, H. E. Scharfman, Hilar granule cells of the mouse dentate gyrus: Effects of age, septotemporal location, strain, and selective deletion of the proapoptotic gene BAX. Brain Struct. Funct. 222, 3147–3161 (2017). 28314928 10.1007/s00429-017-1391-5 PMC5601016

[14] E. Nakahira, S. Yuasa, Neuronal generation, migration, and differentiation in the mouse hippocampal primoridium as revealed by enhanced green fluorescent protein gene transfer by means of in utero electroporation. J. Comp. Neurol. 483, 329–340 (2005). 15682392 10.1002/cne.20441

[15] T. Stuart, A. Butler, P. Hoffman, C. Hafemeister, E. Papalexi, W. M. Mauck III, Y. Hao, M. Stoeckius, P. Smibert, R. Satija, Comprehensive integration of single-cell data. Cell 177, 1888–1902.e21 (2019). 31178118 10.1016/j.cell.2019.05.031 PMC6687398

[16] G. Godbole, A. S. Shetty, A. Roy, L. D’Souza, B. Chen, G. Miyoshi, G. Fishell, S. Tole, Hierarchical genetic interactions between FOXG1 and LHX2 regulate the formation of the cortical hem in the developing telencephalon. Development 145, dev154583 (2018). 29229772 10.1242/dev.154583 PMC5825872

[17] M. Mall, M. S. Kareta, S. Chanda, H. Ahlenius, N. Perotti, B. Zhou, S. D. Grieder, X. Ge, S. Drake, C. Euong Ang, B. M. Walker, T. Vierbuchen, D. R. Fuentes, P. Brennecke, K. R. Nitta, A. Jolma, L. M. Steinmetz, J. Taipale, T. C. Sudhof, M. Wernig, Myt1l safeguards neuronal identity by actively repressing many non-neuronal fates. Nature 544, 245–249 (2017). 28379941 10.1038/nature21722 PMC11348803

[18] Z. Qin, X. Zhou, M. Gomez-Smith, N. R. Pandey, K. F. Lee, D. C. Lagace, J.-C. Béïque, H. H. Chen, LIM domain only 4 (LMO4) regulates calcium-induced calcium release and synaptic plasticity in the hippocampus. J. Neurosci. 32, 4271–4283 (2012). 22442089 10.1523/JNEUROSCI.6271-11.2012 PMC6621222

[19] B. Wang, Y. Zheng, H. Shi, X. Du, Y. Zhang, B. Wei, M. Luo, H. Wang, X. Wu, X. Hua, M. Sun, X. Xu, Zfp462 deficiency causes anxiety-like behaviors with excessive self-grooming in mice. Genes Brain Behav. 16, 296–307 (2017). 27621227 10.1111/gbb.12339

[20] S. Jauhiainen, J. P. Laakkonen, K. Ketola, P. I. Toivanen, T. Nieminen, T. Ninchoji, A. L. Levonen, M. U. Kaikkonen, S. Ylä-Herttuala, Axon guidance-related factor FLRT3 regulates VEGF-signaling and endothelial cell function. Front. Physiol. 10, 224 (2019). 30930791 10.3389/fphys.2019.00224 PMC6423482

[21] V. Danelon, R. Goldner, E. Martinez, I. Gokhman, K. Wang, A. Yaron, T. S. Tran, Modular and distinct plexin-A4/FARP2/Rac1 signaling controls dendrite morphogenesis. J. Neurosci. 40, 5413–5430 (2020). 32499377 10.1523/JNEUROSCI.2730-19.2020 PMC7343331

[22] K. Y. Kwan, M. M. Lam, Z. Krsnik, Y. I. Kawasawa, V. Lefebvre, N. Sestan, SOX5 postmitotically regulates migration, postmigratory differentiation, and projections of subplate and deep-layer neocortical neurons. Proc. Natl. Acad. Sci. U.S.A. 105, 16021–16026 (2008). 18840685 10.1073/pnas.0806791105 PMC2572944

[23] H. Wang, J. Kim, Z. Wang, X.-X. Yan, A. Dean, W. Xu, Crystal structure of human LDB1 in complex with SSBP2. Proc. Natl. Acad. Sci. U.S.A. 117, 1042–1048 (2020). 31892537 10.1073/pnas.1914181117 PMC6969494

[24] M. C. Hernandez, P. J. Andres-Barquin, S. Martinez, A. Bulfone, J. L. Rubenstein, M. A. Israel, ENC-1: A novel mammalian kelch-related gene specifically expressed in the nervous system encodes an actin-binding protein. J. Neurosci. 17, 3038–3051 (1997). 9096139 10.1523/JNEUROSCI.17-09-03038.1997 PMC6573641

[25] L. Shi, N. Muthusamy, D. Smith, C. Bergson, Dynein binds and stimulates axonal motility of the endosome adaptor and NEEP21 family member, calcyon. Int. J. Biochem. Cell Biol. 90, 93–102 (2017). 28734834 10.1016/j.biocel.2017.07.005 PMC5774023

[26] M. D. David, A. Yeramian, M. Dunach, M. Llovera, C. Canti, A. G. de Herreros, J. X. Comella, J. Herreros, Signalling by neurotrophins and hepatocyte growth factor regulates axon morphogenesis by differential beta-catenin phosphorylation. J. Cell Sci. 121, 2718–2730 (2008). 18664491 10.1242/jcs.029660

[27] G. Osaki, S. Mitsui, K. Yuri, The distribution of the seizure-related gene 6 (Sez-6) protein during postnatal development of the mouse forebrain suggests multiple functions for this protein: An analysis using a new antibody. Brain Res. 1386, 58–69 (2011). 21334315 10.1016/j.brainres.2011.02.025

[28] X. Shen, Y. Wei, W. Liu, G. You, S. Tang, Z. Su, M. Du, J. He, J. Zhao, Y. Tian, Y. Zhang, M. Ma, Q. Zhu, H. Yin, A novel circular RNA circITSN2 targets the miR-218-5p/LMO7 axis to promote chicken embryonic myoblast proliferation and differentiation. Front. Cell Dev. Biol. 9, 748844 (2021). 34692701 10.3389/fcell.2021.748844 PMC8526564

[29] M. Co, A. G. Anderson, G. Konopka, FOXP transcription factors in vertebrate brain development, function, and disorders. Wiley Interdiscip. Rev. Dev. Biol. 9, e375 (2020). 31999079 10.1002/wdev.375 PMC8286808

[30] S.-Z. Lin, X.-Y. Zhou, W.-Q. Wang, K. Jiang, Autism with dysphasia accompanied by mental retardation caused by *FOXP1* exon deletion: A case report. World J. Clin. Cases 9, 6858–6866 (2021). 34447835 10.12998/wjcc.v9.i23.6858 PMC8362507

[31] Y. He, X. Lu, F. Qian, D. Liu, R. Chai, H. Li, *Insm1a* is required for zebrafish posterior lateral line development. Front. Mol. Neurosci. 10, 241 (2017). 28824372 10.3389/fnmol.2017.00241 PMC5539400

[32] S. Shinohara, K. Kawasaki, Electrophysiological changes in rat hippocampal pyramidal neurons produced by cholecystokinin octapeptide. Neuroscience 78, 1005–1016 (1997). 9174069 10.1016/s0306-4522(96)00653-7

[33] K. Zybura-Broda, M. Wolder-Gontarek, M. Ambrozek-Latecka, A. Choros, A. Bogusz, J. Wilemska-Dziaduszycka, M. Rylski, HuR (Elavl1) and HuB (Elavl2) Stabilize Matrix Metalloproteinase-9 mRNA During Seizure-Induced Mmp-9 Expression in Neurons. Front. Neurosci. 12, 224 (2018). 29686606 10.3389/fnins.2018.00224 PMC5900018

[34] F. Laub, L. Lei, H. Sumiyoshi, D. Kajimura, C. Dragomir, S. Smaldone, A. C. Puche, T. J. Petros, C. Mason, L. F. Parada, F. Ramirez, Transcription factor KLF7 is important for neuronal morphogenesis in selected regions of the nervous system. Mol. Cell. Biol. 25, 5699–5711 (2005). 15964824 10.1128/MCB.25.13.5699-5711.2005 PMC1157008

[35] X. Ma, Y. Zhou, Y. Chai, X. Wang, X. Huang, Stat3 controls maturation and terminal differentiation in mouse hippocampal neurons. J. Mol. Neurosci. 61, 88–95 (2017). 27785757 10.1007/s12031-016-0820-x

[36] M. J. Galazo, J. G. Emsley, J. D. Macklis, Corticothalamic projection neuron development beyond subtype specification: *Fog2* and intersectional controls regulate intraclass neuronal diversity. Neuron 91, 90–106 (2016). 27321927 10.1016/j.neuron.2016.05.024 PMC5094453

[37] J.-S. Seo, P. Svenningsson, Modulation of ion channels and receptors by p11 (S100A10). Trends Pharmacol. Sci. 41, 487–497 (2020). 32418644 10.1016/j.tips.2020.04.004

[38] Z. Huang, J. Liu, J. Jin, Q. Chen, L. B. E. Shields, Y.-P. Zhang, C. B. Shields, L. Zhou, B. Zhou, P. Yu, Inhibitor of DNA binding 2 promotes axonal growth through upregulation of Neurogenin2. Exp. Neurol. 320, 112966 (2019). 31145898 10.1016/j.expneurol.2019.112966

[39] M. Adachi, P. Y. Lin, H. Pranav, L. M. Monteggia, Postnatal loss of *Mef2c* results in dissociation of effects on synapse number and learning and memory. Biol. Psychiatry 80, 140–148 (2016). 26642739 10.1016/j.biopsych.2015.09.018 PMC4826326

[40] J. Anantha, S. R. Goulding, S. L. Wyatt, R. M. Concannon, L. M. Collins, A. M. Sullivan, G. W. O’Keeffe, STRAP and NME1 mediate the neurite growth-promoting effects of the neurotrophic factor GDF5. iScience 23, 101457 (2020). 32853992 10.1016/j.isci.2020.101457 PMC7452236

[41] M. H. Dominguez, A. E. Ayoub, P. Rakic, POU-III transcription factors (Brn1, Brn2, and Oct6) influence neurogenesis, molecular identity, and migratory destination of upper-layer cells of the cerebral cortex. Cereb. Cortex 23, 2632–2643 (2013). 22892427 10.1093/cercor/bhs252 PMC3792741

[42] A. Miquelajauregui, T. Van de Putte, A. Polyakov, A. Nityanandam, S. Boppana, E. Seuntjens, A. Karabinos, Y. Higashi, D. Huylebroeck, V. Tarabykin, Smad-interacting protein-1 (Zfhx1b) acts upstream of Wnt signaling in the mouse hippocampus and controls its formation. Proc. Natl. Acad. Sci. U.S.A. 104, 12919–12924 (2007). 17644613 10.1073/pnas.0609863104 PMC1929013

[43] K. K. Murai, L. N. Nguyen, F. Irie, Y. Yamaguchi, E. B. Pasquale, Control of hippocampal dendritic spine morphology through ephrin-A3/EphA4 signaling. Nat. Neurosci. 6, 153–160 (2003). 12496762 10.1038/nn994

[44] N. J. Xu, M. Henkemeyer, Ephrin-B3 reverse signaling through Grb4 and cytoskeletal regulators mediates axon pruning. Nat. Neurosci. 12, 268–276 (2009). 19182796 10.1038/nn.2254 PMC2661084

[45] Y. Cheng, N. X. Cawley, T. Yanik, S. R. Murthy, C. Liu, F. Kasikci, D. Abebe, Y. P. Loh, A human carboxypeptidase E/NF-α1 gene mutation in an Alzheimer’s disease patient leads to dementia and depression in mice. Transl. Psychiatry 6, e973 (2016). 27922637 10.1038/tp.2016.237 PMC5315563

[46] C. Pak, T. Danko, V. R. Mirabella, J. Wang, Y. Liu, M. Vangipuram, S. Grieder, X. Zhang, T. Ward, Y. A. Huang, K. Jin, P. Dexheimer, E. Bardes, A. Mitelpunkt, J. Ma, M. McLachlan, J. C. Moore, P. Qu, C. Purmann, J. L. Dage, B. J. Swanson, A. E. Urban, B. J. Aronow, Z. P. Pang, D. F. Levinson, M. Wernig, T. C. Sudhof, Cross-platform validation of neurotransmitter release impairments in schizophrenia patient-derived *NRXN1*-mutant neurons. Proc. Natl. Acad. Sci. U.S.A. 118, e2025598118 (2021). 34035170 10.1073/pnas.2025598118 PMC8179243

[47] O. Yarishkin, D. Y. Lee, E. Kim, C. H. Cho, J. H. Choi, C. J. Lee, E. M. Hwang, J.-Y. Park, TWIK-1 contributes to the intrinsic excitability of dentate granule cells in mouse hippocampus. Mol. Brain 7, 80 (2014). 25406588 10.1186/s13041-014-0080-z PMC4240835

[48] F. Bedogni, R. D. Hodge, G. E. Elsen, B. R. Nelson, R. A. Daza, R. P. Beyer, T. K. Bammler, J. L. Rubenstein, R. F. Hevner, Tbr1 regulates regional and laminar identity of postmitotic neurons in developing neocortex. Proc. Natl. Acad. Sci. U.S.A. 107, 13129–13134 (2010). 20615956 10.1073/pnas.1002285107 PMC2919950

[49] T. Andersson, E. Sodersten, J. K. Duckworth, A. Cascante, N. Fritz, P. Sacchetti, I. Cervenka, V. Bryja, O. Hermanson, CXXC5 is a novel BMP4-regulated modulator of Wnt signaling in neural stem cells. J. Biol. Chem. 284, 3672–3681 (2009). 19001364 10.1074/jbc.M808119200

[50] K. Nakajima, M. Inagawa, C. Uchida, K. Okada, S. Tane, M. Kojima, M. Kubota, M. Noda, S. Ogawa, H. Shirato, M. Sato, R. Suzuki-Migishima, T. Hino, Y. Satoh, M. Kitagawa, T. Takeuchi, Coordinated regulation of differentiation and proliferation of embryonic cardiomyocytes by a jumonji (Jarid2)-cyclin D1 pathway. Development 138, 1771–1782 (2011). 21447557 10.1242/dev.059295

[51] F. Cargnin, J. S. Kwon, S. Katzman, B. Chen, J. W. Lee, S.-K. Lee, FOXG1 orchestrates neocortical organization and cortico-cortical connections. Neuron 100, 1083–1096.e5 (2018). 30392794 10.1016/j.neuron.2018.10.016 PMC6428593

[52] Z. Xie, X. Ma, W. Ji, G. Zhou, Y. Lu, Z. Xiang, Y. X. Wang, L. Zhang, Y. Hu, Y. Q. Ding, W. J. Zhang, Zbtb20 is essential for the specification of CA1 field identity in the developing hippocampus. Proc. Natl. Acad. Sci. U.S.A. 107, 6510–6515 (2010). 20308569 10.1073/pnas.0912315107 PMC2851958

[53] B. Liu, K. Zhou, X. Wu, C. Zhao, Foxg1 deletion impairs the development of the epithalamus. Mol. Brain 11, 5 (2018). 29394901 10.1186/s13041-018-0350-2 PMC5797387

[54] J. V. Nielsen, M. Thomassen, K. Møllgard, J. Noraberg, N. A. Jensen, Zbtb20 defines a hippocampal neuronal identity through direct repression of genes that control projection neuron development in the isocortex. Cereb. Cortex 24, 1216–1229 (2014). 23283686 10.1093/cercor/bhs400

[55] H. C. Chuang, T. N. Huang, Y. P. Hsueh, T-brain-1--A potential master regulator in autism spectrum disorders. Autism Res. 8, 412–426 (2015). 25600067 10.1002/aur.1456

[56] S. Tole, C. Christian, E. A. Grove, Early specification and autonomous development of cortical fields in the mouse hippocampus. Development 124, 4959–4970 (1997). 9362459 10.1242/dev.124.24.4959

[57] M. M. Haznedar, M. S. Buchsbaum, T. C. Wei, P. R. Hof, C. Cartwright, C. A. Bienstock, E. Hollander, Limbic circuitry in patients with autism spectrum disorders studied with positron emission tomography and magnetic resonance imaging. Am. J. Psychiatry 157, 1994–2001 (2000). 11097966 10.1176/appi.ajp.157.12.1994

[58] D. Wegrzyn, G. Juckel, A. Faissner, Structural and functional deviations of the hippocampus in Schizophrenia and Schizophrenia animal models. Int. J. Mol. Sci. 23, 5482 (2022). 35628292 10.3390/ijms23105482 PMC9143100

[59] J. Mariani, G. Coppola, P. Zhang, A. Abyzov, L. Provini, L. Tomasini, M. Amenduni, A. Szekely, D. Palejev, M. Wilson, M. Gerstein, E. L. Grigorenko, K. Chawarska, K. A. Pelphrey, J. R. Howe, F. M. Vaccarino, FOXG1-dependent dysregulation of GABA/glutamate neuron differentiation in autism spectrum disorders. Cell 162, 375–390 (2015). 26186191 10.1016/j.cell.2015.06.034 PMC4519016

[60] R. Zhang, H. He, B. Yuan, Z. Wu, X. Wang, Y. Du, Y. Chen, Z. Qiu, An intronic variant of *CHD7* identified in autism patients interferes with neuronal differentiation and development. Neurosci. Bull. 37, 1091–1106 (2021). 33948885 10.1007/s12264-021-00685-w PMC8353028

[61] L. Qin, K. Ma, Z.-J. Wang, Z. Hu, E. Matas, J. Wei, Z. Yan, Publisher correction: Social deficits in Shank3-deficient mouse models of autism are rescued by histone deacetylase (HDAC) inhibition. Nat. Neurosci. 21, 1139 (2018). 10.1038/s41593-018-0110-8 PMC5876144 29531362

[62] J. Li, L. Zhu, F. Guan, Z. Yan, D. Liu, W. Han, T. Chen, Relationship between schizophrenia and changes in the expression of the long non-coding RNAs Meg3, Miat, Neat1 and Neat2. J. Psychiatr. Res. 106, 22–30 (2018). 30243133 10.1016/j.jpsychires.2018.09.005

[63] S. Wang, D. Wang, X. Cai, Q. Wu, Y. Han, Identification of the *ZEB2* gene as a potential target for epilepsy therapy and the association between rs10496964 and *ZEB2* expression. J. Int. Med. Res. 48, 300060520980527 (2020). 33870748 10.1177/0300060520980527 PMC8061191

[64] L. Smith, N. Singhal, C. M. El Achkar, G. Truglio, B. Rosen Sheidley, J. Sullivan, A. Poduri, *PCDH19*-related epilepsy is associated with a broad neurodevelopmental spectrum. Epilepsia 59, 679–689 (2018). 29377098 10.1111/epi.14003 PMC6264912

[65] G. C. Korenke, B. Schulte, S. Biskup, J. Neidhardt, M. Owczarek-Lipska, A novel de novo frameshift mutation in the *BCL11A* gene in a patient with intellectual disability syndrome and epilepsy. Mol. Syndromol. 11, 135–140 (2020). 32903878 10.1159/000508566 PMC7445578

[66] M. Su, J. Liu, B. Yu, K. Zhou, C. Sun, M. Yang, C. Zhao, Loss of *Calretinin* in L5a impairs the formation of the barrel cortex leading to abnormal whisker-mediated behaviors. Mol. Brain 14, 67 (2021). 33845857 10.1186/s13041-021-00775-w PMC8042711

[67] A. G. Anderson, A. Kulkarni, M. Harper, G. Konopka, Single-cell analysis of Foxp1-driven mechanisms essential for striatal development. Cell Rep. 30, 3051–3066.e7 (2020). 32130906 10.1016/j.celrep.2020.02.030 PMC7137930

