# Supplementary material for: FOXG1 drives transcriptomic networks to specify principal neuron subtypes during the development of the medial pallium
Source: Sci Adv. 2023 Feb 15;9(7):eade2441. doi: 10.1126/sciadv.ade2441 (PMC9931217; doi:10.1126/sciadv.ade2441)
Supplement: Supplementary file 1 — Figs. S1 to S12 Legends for tables S1 to S6 [file sciadv.ade2441_sm.pdf]

Science Advances
AAAS

Supplementary Materials for

FOXG1 drives transcriptomic networks to specify principal neuron subtypes during the development of the medial pallium

Ru Ba *et al.*

Corresponding author: Chunjie Zhao, zhaocj@seu.edu.cn

Sci. Adv. **9**, eade2441 (2023)
DOI: 10.1126/sciadv.ade2441

The PDF file includes:

Figs. S1 to S12
Legends for tables S1 to S6

Other Supplementary Material for this manuscript includes the following:

Tables S1 to S6

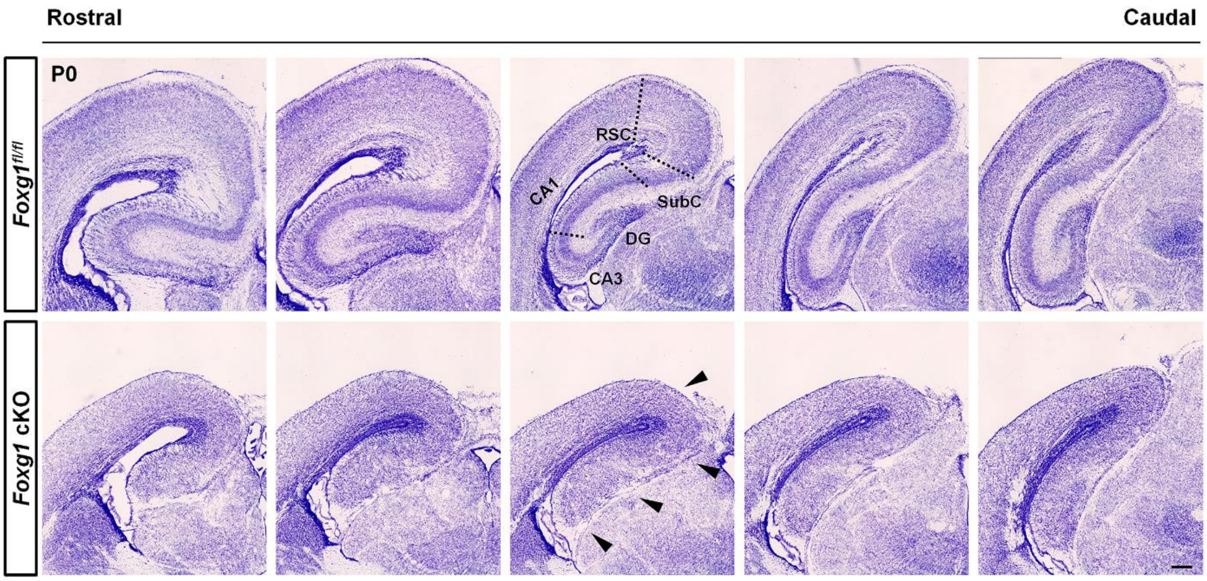

fig. S1. Postmitotic deletion of *Foxg1* disrupts MP structures.

(A) Nissl staining of serial coronal brain sections at P0 showing that the typical features of the RSC, SubC, CA1-CA3 and DG disappeared in *Foxg1* cKO mice.

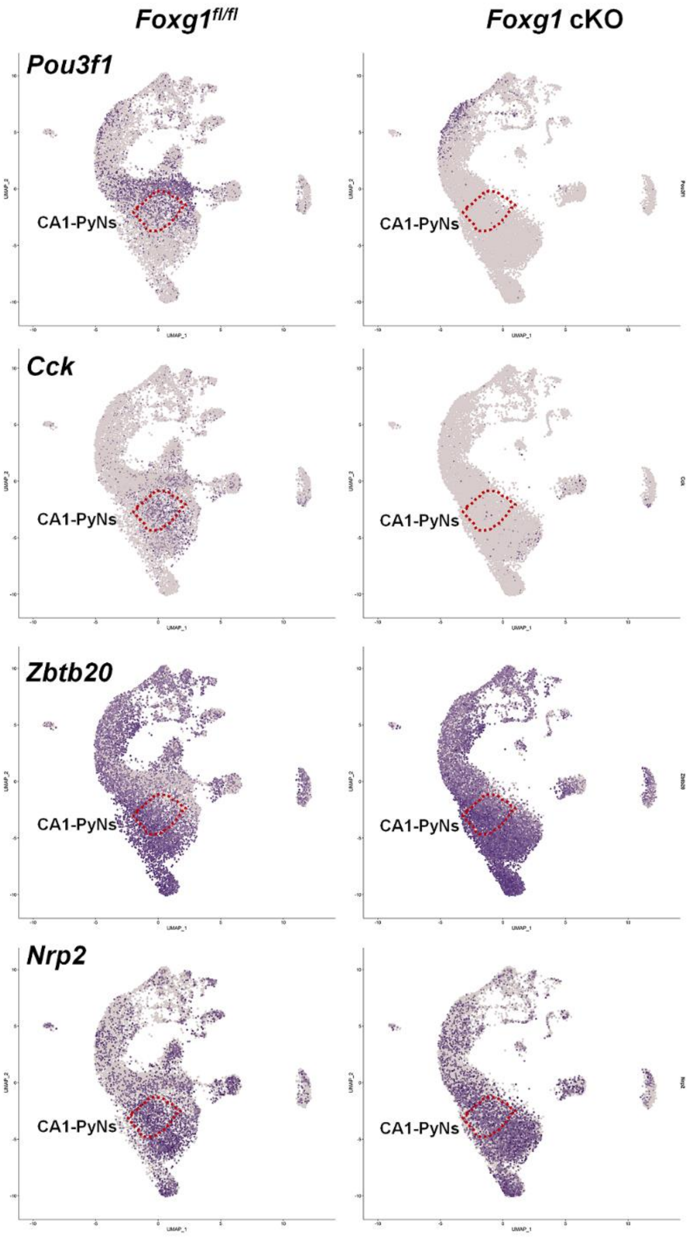

fig. S2. Expression of gene markers specific to CA1-PyNs and markers coexpressed between CA1-PyNs and CA3-PyNs.

(A) UMAP plots showing the expression of gene markers, *Pou3f1*, and *Cck*, which are specific to CA1-PyNs, and markers, *Zbtb20*, and *Nrp2*, which are coexpressed between CA1-PyNs and CA3-PyNs in control and *Foxg1* cKO mice. The color key indicates expression levels (purple, high; gray, low).

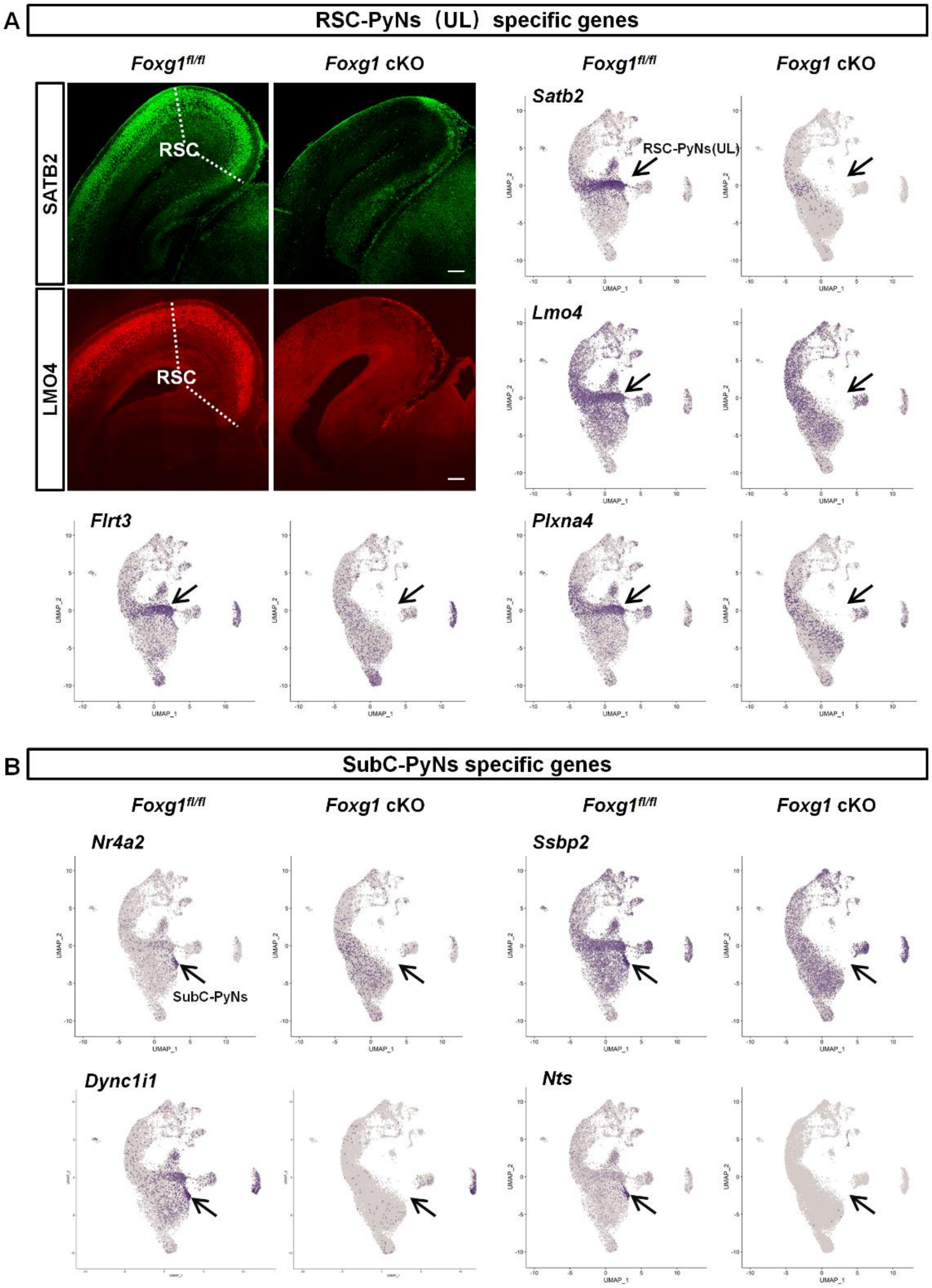

fig. S3. Identification of RSC-PyNs(UL) and SubC-PyNs specific genes by scRNA-seq between the two genotypes.

(A) Immunostaining against SATB2 and LMO4 at P0, showing loss of SATB2 and LMO4 in the RSC-PyNs (UL) in *Foxg1* cKO mice. UMAP plots showing the expression of *Satb2*, *Lmo4*,

Flrt3, and *Plxna4* in control and *Foxg1* cKO mice. The color key indicates expression levels (purple, high; gray, low).

(B) UMAP plots showing the expression of SubC-PyNs-specific genes *Nr4a2*, *Ssbp2*, *Dync1i1*, and *Nts* in control and *Foxg1* cKO mice.

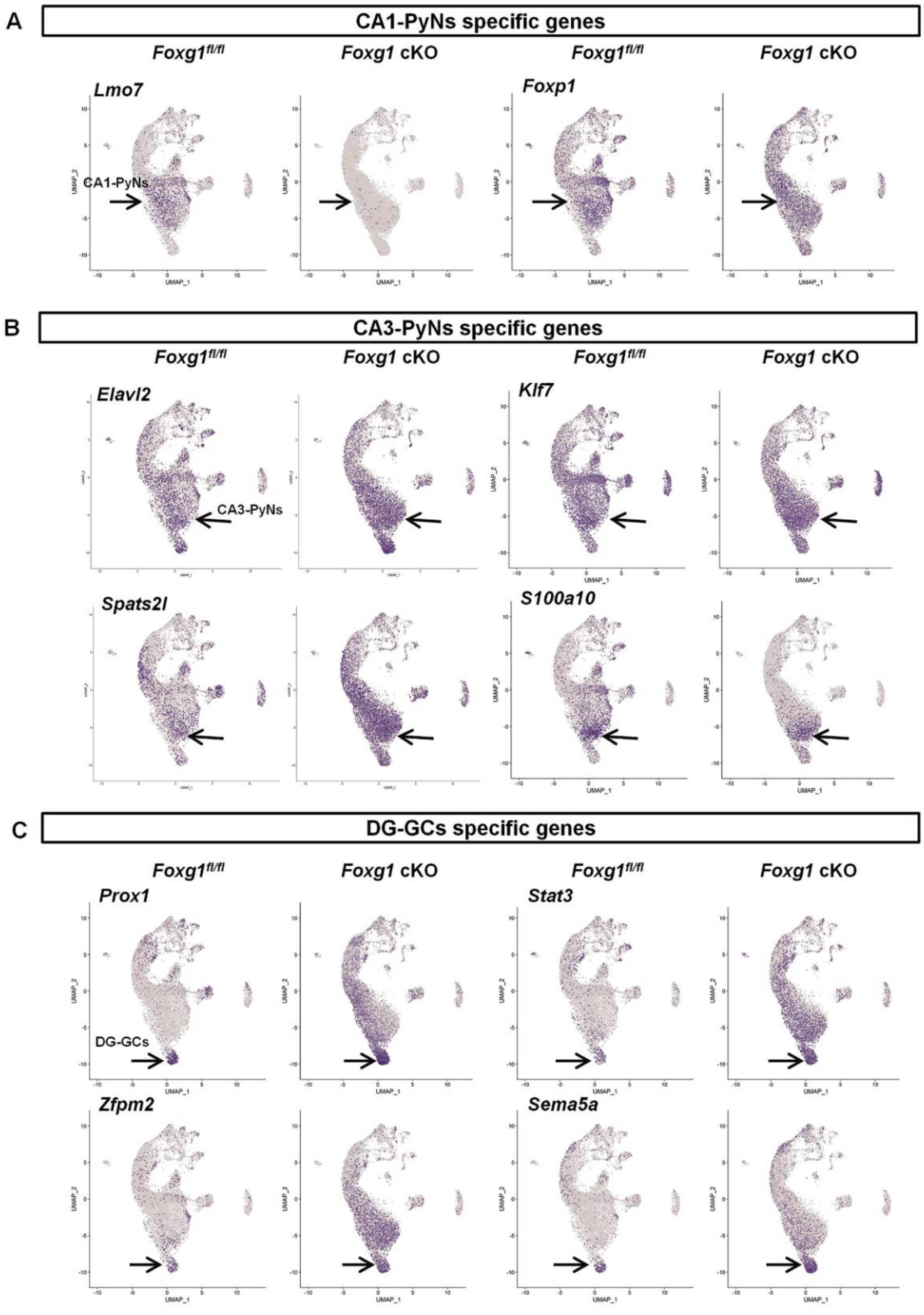

fig. S4. Identification of hippocampal neuron subtype-specific genes by scRNA-seq between the two genotypes.

(A) UMAP plots showing the expression of the CA1-PyN-specific genes *Lmo7* and *Foxp1* in control and *Foxg1* cKO mice.

(B) UMAP plots showing the expression of the CA3-PyN-specific genes *Elavl2*, *Klf7*, *S100a10*, and *Spats2l* in control and *Foxg1* cKO mice.

(C) UMAP plots showing the expression of the DG-GC-specific genes *Prox1*, *Stat3*, *Zfpm2* and *Sema5a* in control and *Foxg1* cKO mice.

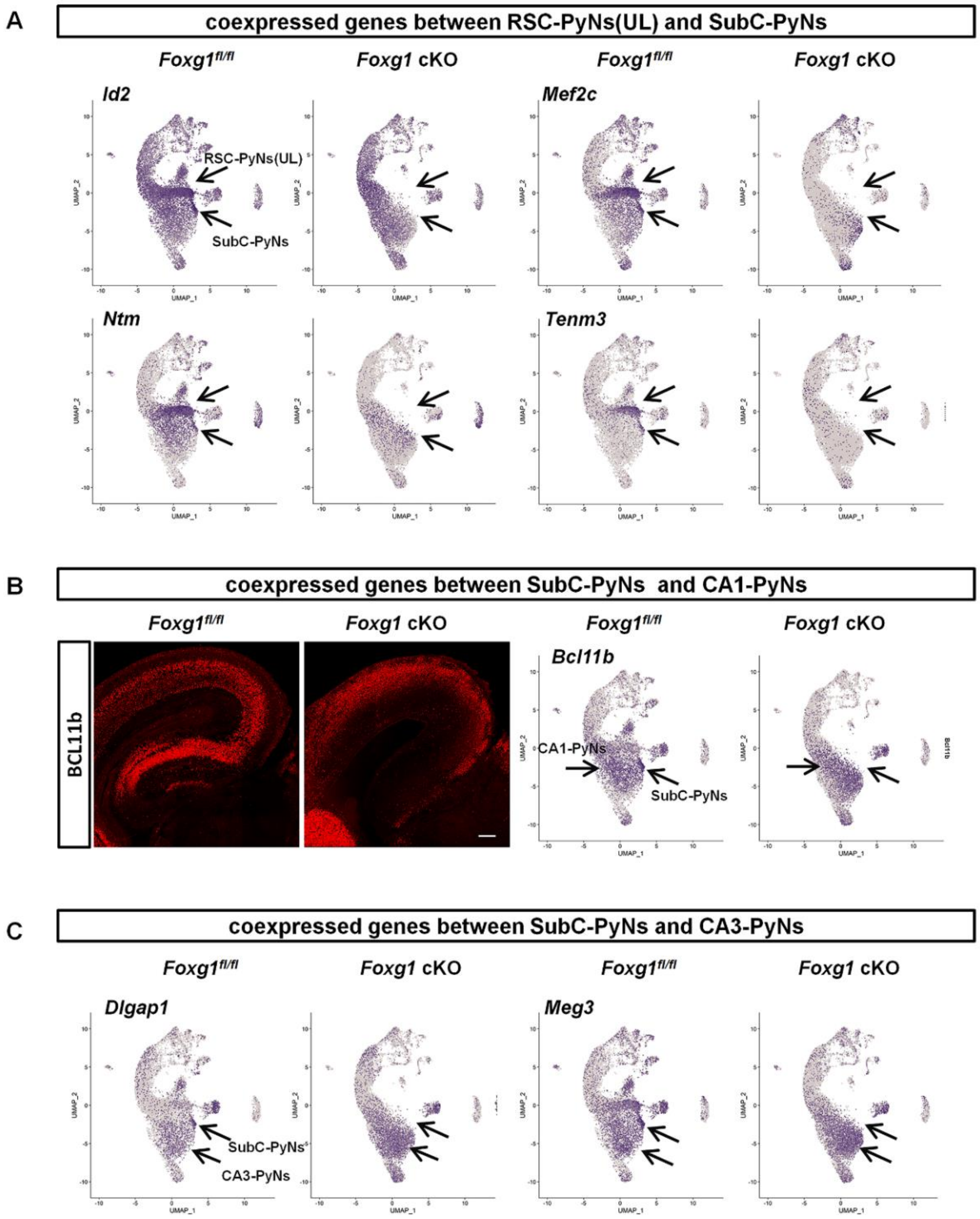

fig. S5. Identification of coexpressed genes between SubC-PyNs and RSC-PyNs (UL), SubC-PyNs and CA1-PyNs, and SubC-PyNs and CA3-PyNs by scRNA-seq between the two genotypes.

(A) UMAP plots showing the expression of coexpressed genes between SubC-PyNs and RSC-PyNs (UL) *Id2*, *Mef2c*, *Ntm*, and *Tenm3* in control and *Foxg1* cKO mice.

(B) Immunostaining against BCL11B at P0, showing Bcl11B downregulated in *Foxg1* cKO MP. UMAP plots showing *Bcl11b* downregulation in the MP but upregulation in the neocortex in *Foxg1* cKO mice.

(C) UMAP plots showing the expression of the coexpressed genes *Dlgap1* and *Meg3* between SubC-PyNs and CA3-PyNs in control and *Foxg1* cKO mice.

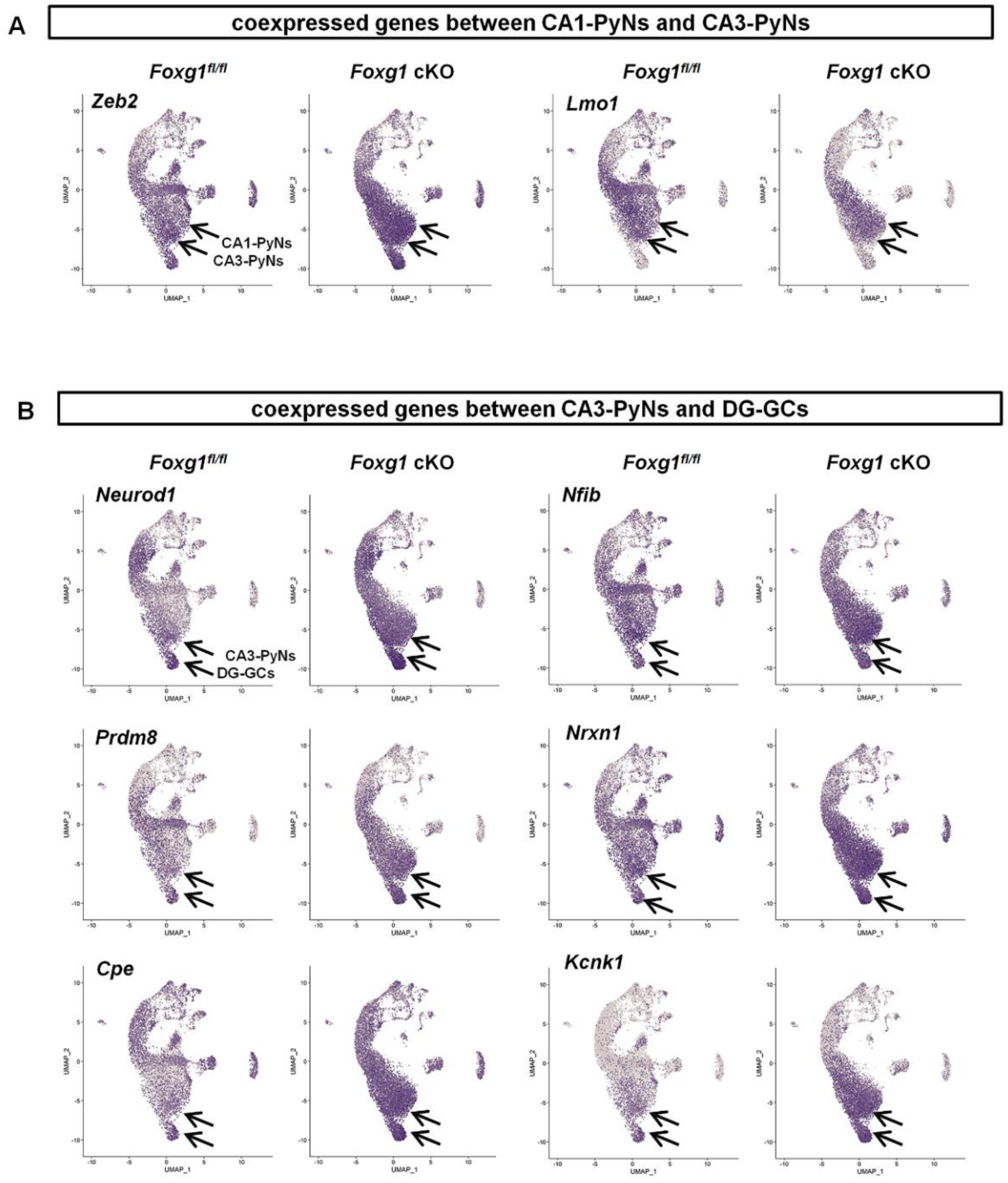

fig. S6. Identification of coexpressed genes between CA3-PyNs and CA1-PyNs and CA3-PyNs and DG-GCs by scRNA-seq between the two genotypes.

(A) UMAP plots showing the expression of the coexpressed genes *Zeb2* and *Lmo1* between CA3-PyNs and CA1-PyNs in control and *Foxg1* cKO mice.

(B) UMAP plots showing the expression of the coexpressed genes *Neurod1*, *Nfib*, *Prdm8*, *Nrxn1*, *Cpe*, and *Kcnk1* between CA3-PyNs and DG-GCs in control and *Foxg1* cKO mice.

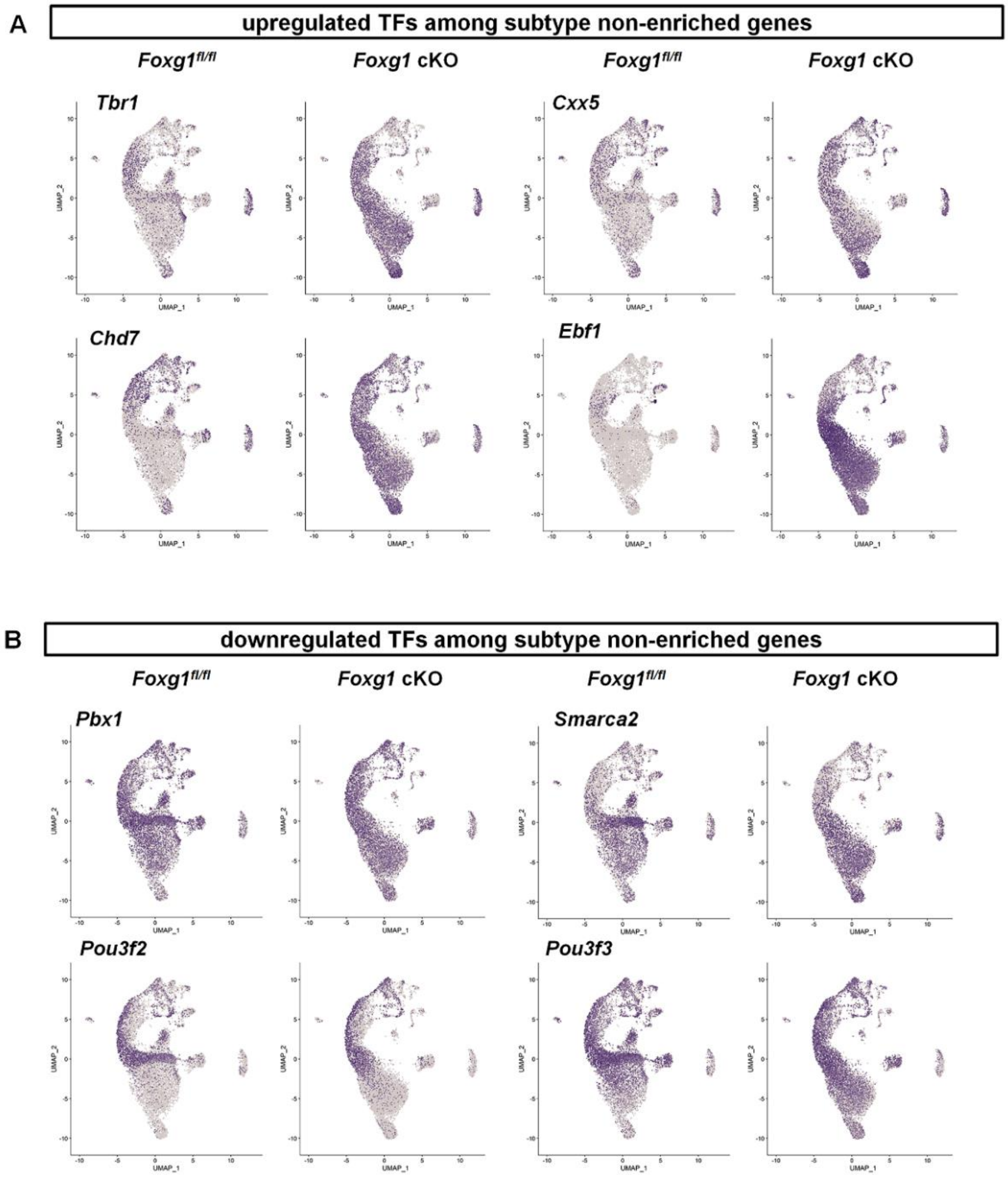

fig. S7. Identification of subtype-nonenriched genes by scRNA-seq between the two genotypes.

(A) UMAP plots showing the upregulation of subtype-nonenriched genes *Tbr1*, *Cxx5*, *Chd7*, and *Ebf1* in the *Foxg1* cKO MP.

(B) UMAP plots showing the downregulation of subtype-nonenriched genes *Pbx1*, *Smarca2*, *Pou3f2*, and *Pou3f3* in the *Foxg1* cKO MP.

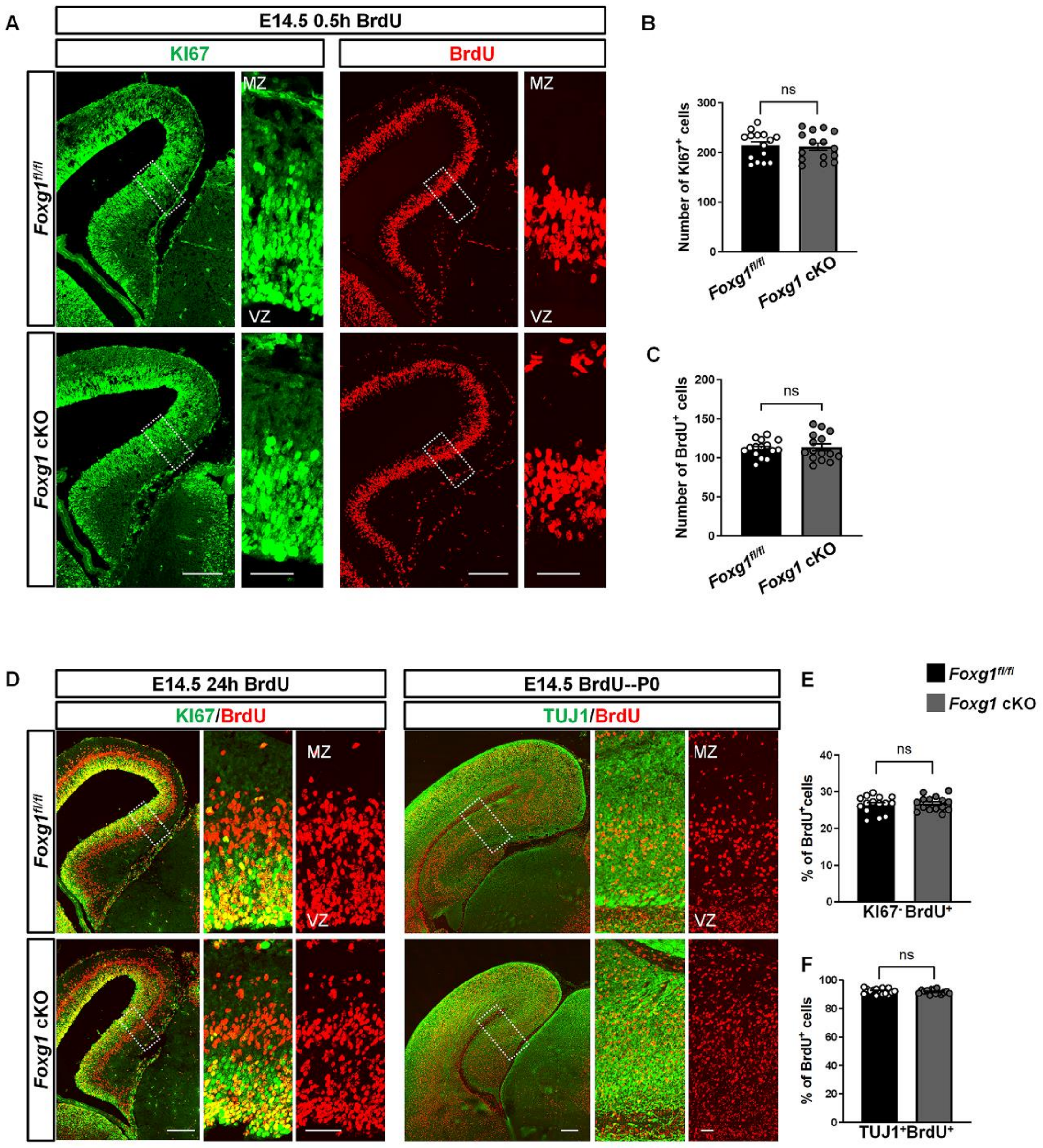

fig. S8. Postmitotic deletion of *Foxg1* does not alter the proliferation and differentiation in the MP.

(A) Immunostaining against cell cycle marker KI67 and BrdU after 0.5h pulse at E14.5 showing the proliferation is comparable between the control and *Foxg1* cKO mice.

(B) Quantification analysis of the number of $KI67^+$ cells at E14.5 showing no significant difference between the control and *Foxg1* cKO mice.

(C) Quantification analysis of the number of $BrdU^+$ cells at E14.5 showing no significant differences between the control and *Foxg1* cKO mice.

(D) Immunostaining against KI67 and BrdU after 24h BrdU injection at E14.5, and immunostaining against TUJ1 and BrdU at P0 after BrdU injection at E14.5 showing that the differentiation is not changed in *Foxg1* cKO mice.

(E) Quantification analysis of the ratio of $KI67^-BrdU^+$ cells/ $BrdU^+$ cells showing cell cycle exit is not altered in *Foxg1* cKO mice.

(F) Quantification analysis of the ratio of $TUJ1^+BrdU^+$ cells/ $BrdU^+$ cells showing no significant differences between the control and the *Foxg1* cKO mice.

Data are presented as the mean± SEM, B, C, E, F, unpaired Student's *t* test, ns: not significant.

Scale bar=200μm in overview of A, D, the magnification of A, D, scale bar=50μm.

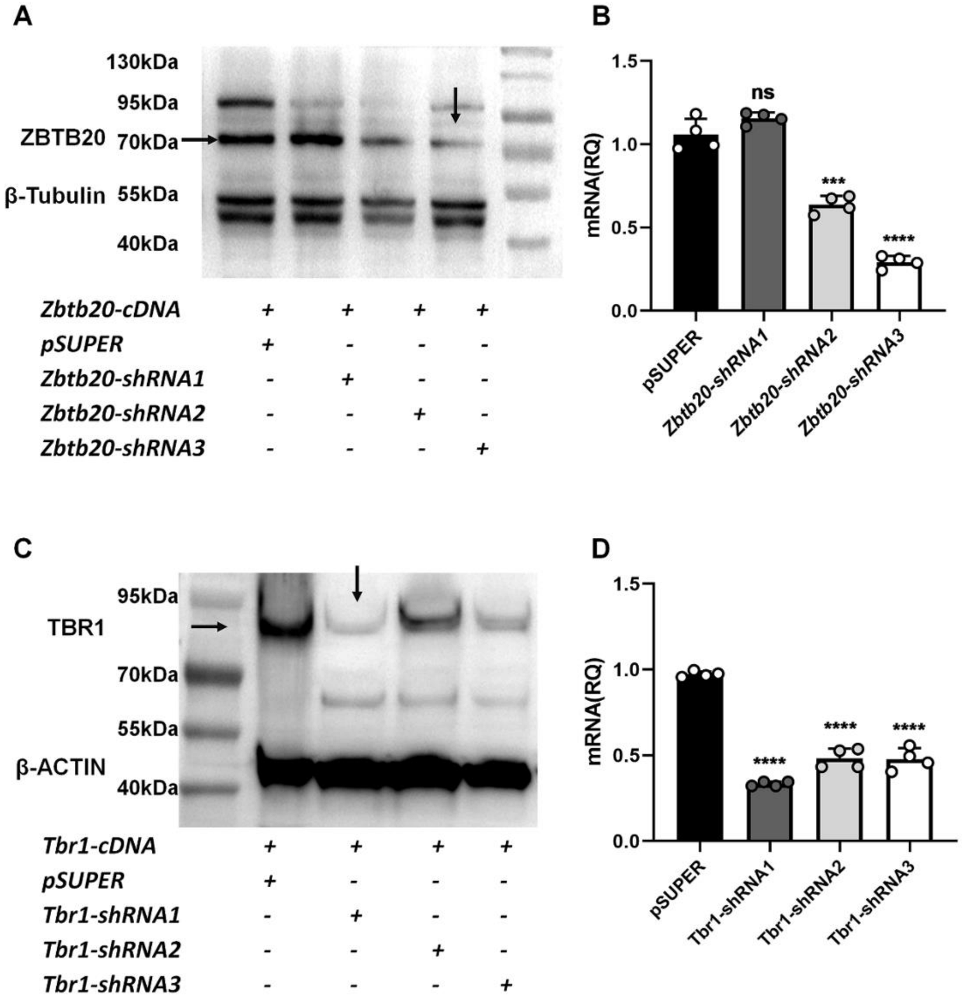

fig. S9. Assessment of ZBTB20 and TBR1 knockdown efficiency.

(A) Western blots of N2A cells transfected with *Zbtb20-shRNA* showing efficient ZBTB20 knockdown by *Zbtb20-shRNA3*.

(B) qPCR of N2A cell samples transfected with *Zbtb20-shRNA* showing efficient ZBTB20 knockdown by *Zbtb20-shRNA3*.

(C) Western blots of N2A cells transfected with *Tbr1-shRNA* showing efficient TBR1 knockdown by *Tbr1-shRNA1*.

(D) qPCR of N2A cell samples transfected with *Tbr1-shRNA* showing efficient TBR1 knockdown by *Tbr1-shRNA1*.

Data are presented as the mean± SEM, B, D unpaired Student's *t* test, ***$P < 0.001$, ****$P < 0.0001$, ns: not significant.

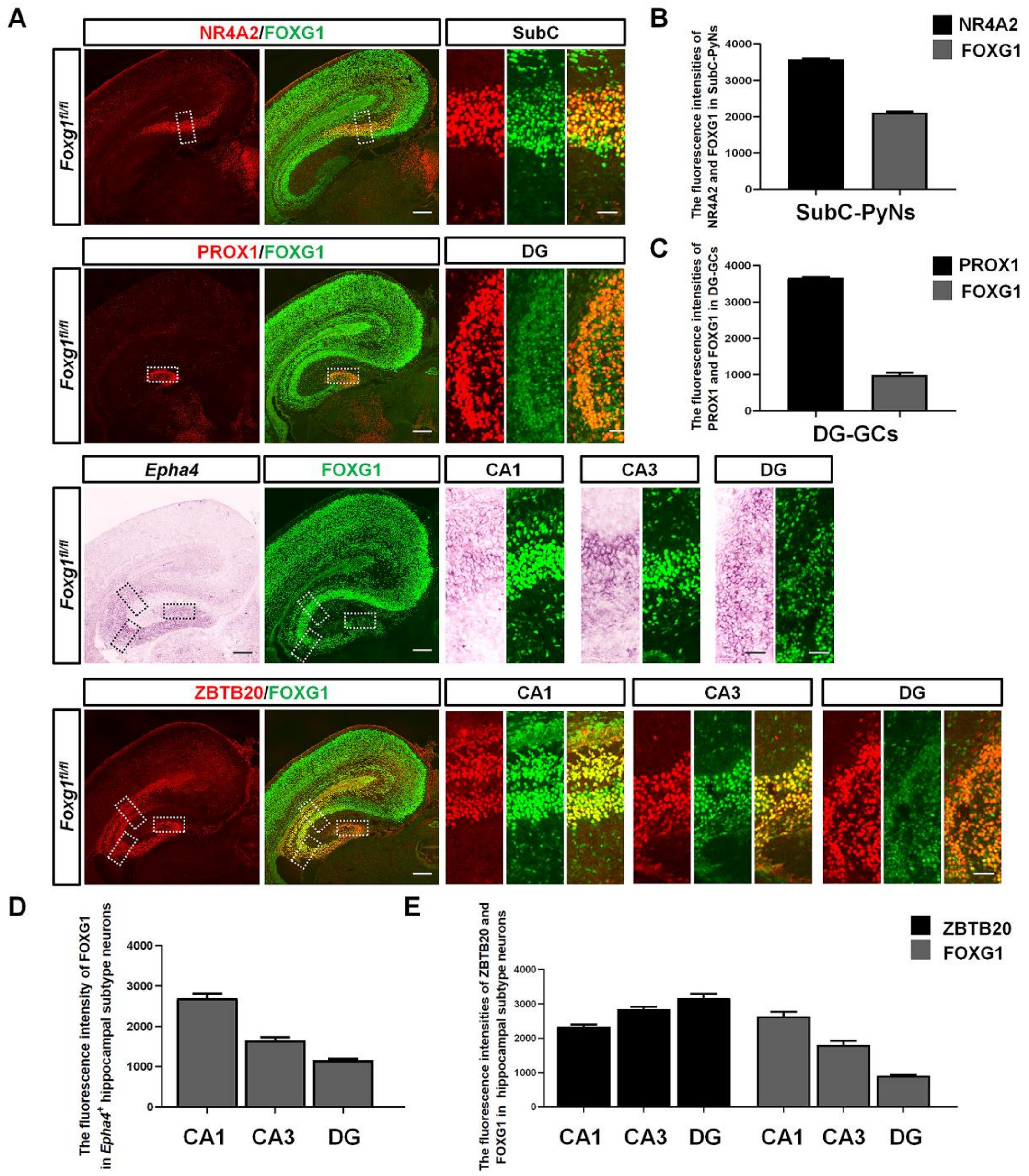

fig. S10. Assessment of the expression levels of FOXG1 in MP different neuron subtypes.

(A) Immunostaining against FOXG1 with NR4A2, PROX1 and ZBTB20, and *Epha4 in situ* hybridization at P0 in control mice.

(B) Quantification analysis of the fluorescence intensities of NR4A2 and FOXG1 showing high expression level of FOXG1 in SubC-PyNs.

(C) Quantification analysis of the fluorescence intensities of PROX1 and FOXG1 showing low expression level of FOXG1 in DG-GCs.

(D) Quantification analysis of the fluorescence intensities of FOXG1 showing that the expression of FOXG1 exhibits a $CA1^{high}$-DG^{low} pattern which is complementary to that of *Epha4*.

(E) Quantification analysis of the fluorescence intensities of ZBTB20 and FOXG1 showing that the expression of FOXG1 exhibits a $CA1^{high}$-DG^{low} pattern which is complementary to that of ZBTB20.

Data are presented as the mean± SEM, B, C, D, E

Scale bar=200μm in overview of A, the magnification of A, scale bar=50μm.

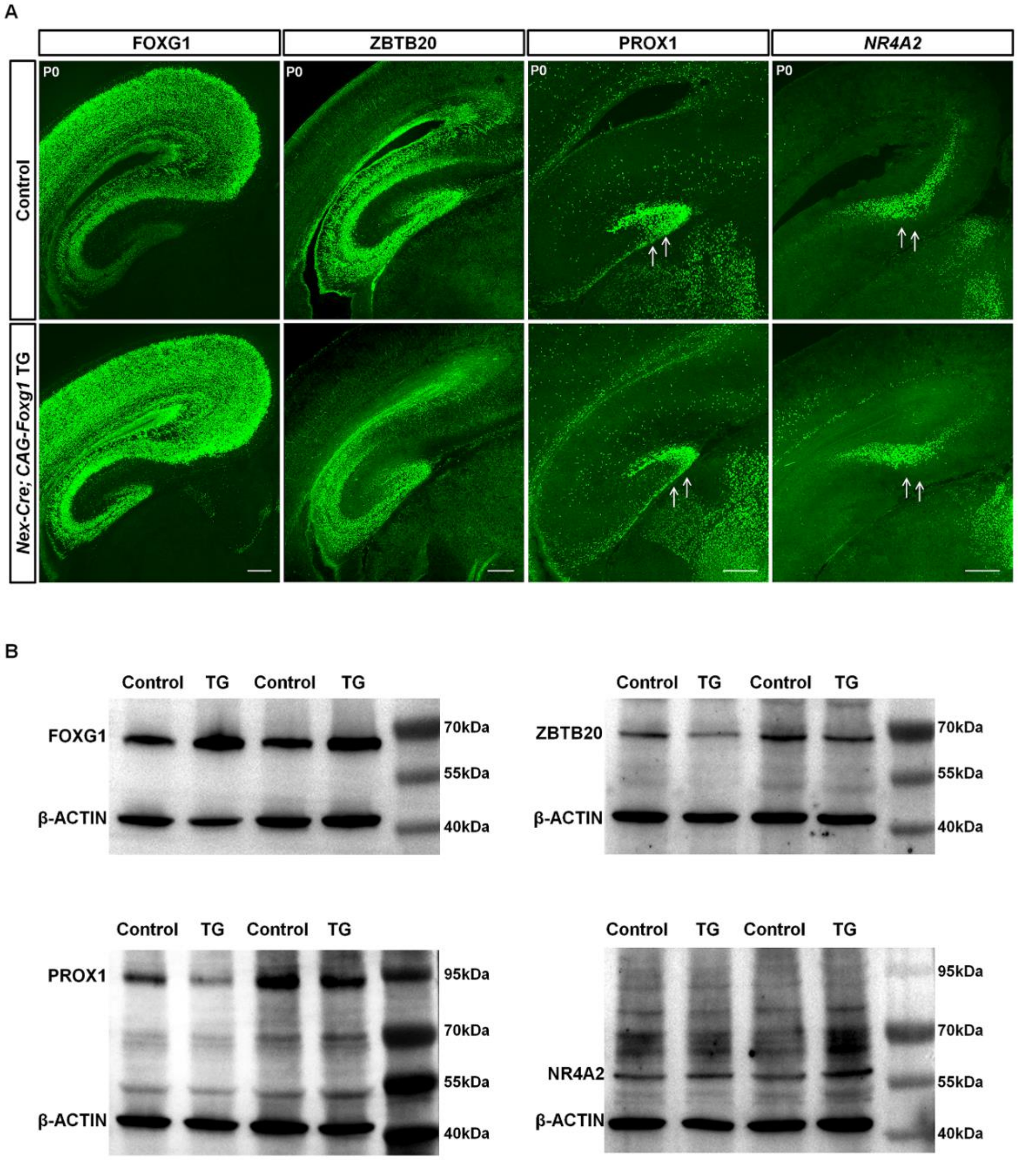

fig. S11. Overexpression of FOXG1 is capable to repress *Zbtb20*, *Prox1*, and promote *Nr4a2*.

(A) immunostaining against FOXG1, ZBTB20, PROX1, and NR4A2 in P0 *Nex-Cre; CAG-loxp-stop-loxp-Foxg1-IRES-EGFP* mice showing an obvious decrease in the expression levels of ZBTB20 and PROX1 and a slightly increased NR4A2 level in the *Foxg1* overexpressed MP.

(B) Western blots at P0 confirm the downregulation of ZBTB20 and PROX1, the upregulation of NR4A2 after *Foxg1* overexpression in *Nex-Cre; CAG-loxp-stop-loxp-Foxg1-IRES-EGFP* mice.

Scale bar=200μm in overview of A.

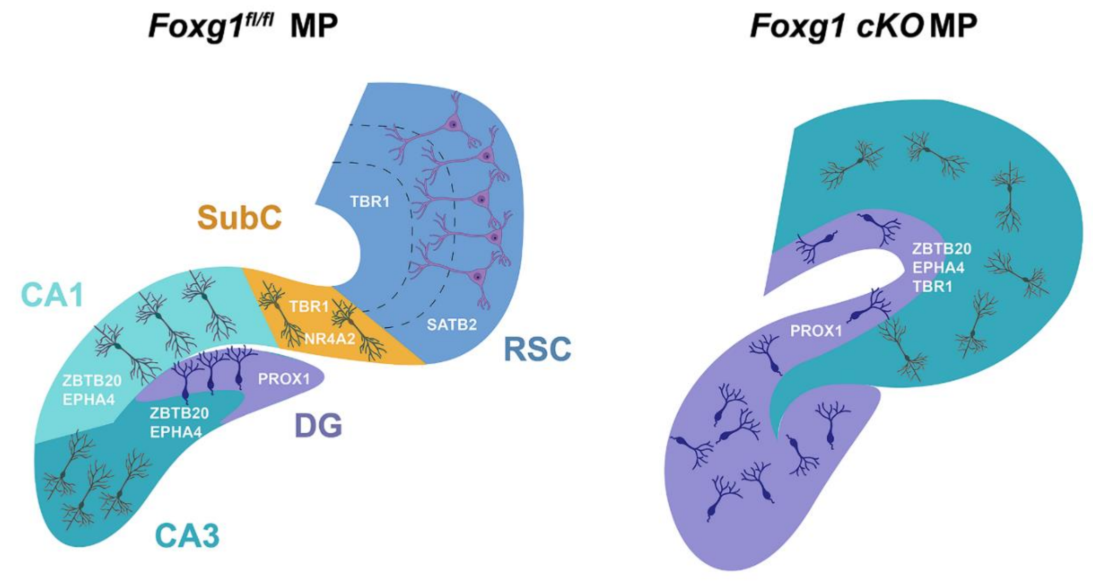

fig. S12. Summary model of the FOXG1-regulated transcriptional mechanism controlling MP principal neuron subtype specification.

Supplementary tables

****Note: All supplementary tables have been provided as separate files.***

table S1: MP 7 subtypes enriched genes

table S2: MP-DEGs

table S3: MP-DEGs vs FOXG1 candidate target genes

table S4: The antibodies for immunofluorescence and western blotting analyses

table S5: The primers for *in situ* probes and qPCR

table S6: List of construction carrying point mutation in FOXG1-binding loci
