# Supplementary material for: FOXG1 drives transcriptomic networks to specify principal neuron subtypes during the development of the medial pallium
Source: Sci Adv. 2023 Feb 15;9(7):eade2441. doi: 10.1126/sciadv.ade2441 (PMC9931217; doi:10.1126/sciadv.ade2441)
Supplement: Supplementary file 2 — Tables S1 to S6 [file sciadv.ade2441_tables_s1_to_s6.zip › sciadv.ade2441_tables_s4_to_s6.docx]

**Table S4. The antibodies for immunofluorescence and western blotting analyses**

| Rabbit anti-FOXG1 | Abcam | Cat. #AB18259; RRID: AB_732415 |
| --- | --- | --- |
| Rabbit anti-CALRETININ | Millipore | Cat. # AB5054; RRID: AB_2068506 |
| Mouse anti-SYNAPTOPORIN | Santa Cruz | Cat. # sc-376761; RRID: AB_564017 |
| Rabbit anti-ZBTB20 | Synaptic System | Cat. #262003; RRID: AB_2739245 |
| Mouse anti-ZBTB20 | Santa Cruz | Cat. #sc-515370; RRID: AB_2924763 |
| Chicken anti-GFP | Abcam | Cat. #ab13970; RRID: AB_300798 |
| Goat anti-PROX1 | R&D | Cat. #AF2727; RRID: AB_2170716 |
| Mouse anti-NR4A2 | Abcam | Cat. #ab41917; RRID: AB_776887 |
| Rat anti-BCL11B | Abcam | Cat. #ab18462; RRID: AB_2064130 |
| Guinea pig anti-TBR1 | Synaptic System | Cat. #328005; RRID: AB_2620072 |
| Rabbit anti-POU3F1 | GeneTex | Cat. #GTX134063; RRID: AB_2887197 |
| Rabbit anti-FOXP1 | Abcam | Cat. #ab16645; RRID: AB_732428 |
| Mouse anti-SATB2 | Santa Cruz | Cat. # sc-81376; RRID: AB_1129287 |
| Goat anti-LMO4 | Santa Cruz | Cat. # sc-11122; RRID: AB_648429 |
| Rabbit anti-KI67 | Abcam | Cat. #ab16667; RRID: AB_302459 |
| Rat anti-BrdU | Abcam | Cat. #ab6326; RRID: AB_305426 |
| Mouse anti-TUJ1 | Abcam | Cat. #ab78078; RRID: AB_2256751 |
| Mouse anti-PAX6 | Santa Cruz | Cat. #sc-81649; RRID: AB_1127044 |
| Rabbit anti-CASPASE3 | Millipore | Cat. #AB3623; RRID: AB_303959 |
| Rabbit anti-GAPDH | Abcam | Cat. #2251-1; RRID:AB_126717 |
| Rabbit anti-β-ACTIN | Cell signaling Technology | Cat. #4967; RRID: AB_330288 |
| Rabbit anti-β-TUBULIN | Cell signaling Technology | Cat. #15115; RRID: AB_2798712 |
| anti-digoxigenin-alkaline phosphatase | Roche | Cat. #11093274910; RRID: AB_514497 |
| Rabbit anti-IgG | Millipore | Cat. #12-370; RRID: 145841 |

**Table S5. The primers for *in situ* probes and qPCR**

| Mouse *Nef3* (*in situ*) | F: 5’- GATGAAGGTGAGCAGGAAGAA-3’ |
| --- | --- |
|  | R: 5’- CTGGGTGACTTCCTTGACTATG-3’ |
| Mouse *Fn1* (*in situ*) | F: 5’- CCCTTACAGTTCCAAGTTCCTG-3’ |
|  | R: 5’- AAAGGCTTAAGGGTGAAAGGAC-3’ |
| Mouse *Mpped1* (*in situ*) | F: 5’- GACCAGCCAGCAACGAGT-3’ |
|  | R: 5’- GGAACCCTCGGAAGGAAA-3’ |
| Mouse *Grik4* (*in situ*) | F: 5’- GGAAGGCAATGACCGATATGA-3’ |
|  | R: 5’- GTACTGGTTGACCAGGAGATTAC-3’ |
| Mouse *EphA4* (*in situ*) | F: 5’- CGCGAATGAAGTTACTTT A-3’ |
|  | R: 5’- TATCAGGAAACTGGGCTAGAT-3’ |
| Mouse *Zbtb20* (ChIP-qPCR) | F: 5’- TTCCTCCAGAAGCCTGTCTA-3’ |
|  | R: 5’- CGGCAACCTCTTGCATTTG-3’ |
| Mouse *Prox1*(ChIP-qPCR) | F: 5’- TCTAGGCTGATGGCGTTGTGCTG-3’ |
|  | R: 5’- ACACTGGCTGCTCTGGAAGGGAA-3’ |
| Mouse *Epha4*(ChIP-qPCR) | F: 5’- GTGTCTAACTCACTGGGTTCTG-3’ |
|  | R: 5’- CCCTTCCACAAAGGCAACTA-3’ |
| Mouse *Nr4a2* (ChIP-qPCR) | F: 5’- AGCCAGCGGGGCCGGAGAGAGGGAC-3’ |
|  | R: 5’- TGCATGTTGGCAGCAGCAGCTCGA-3’ |
| Mouse *Tbr1*(ChIP-qPCR) | F: 5’- GCTAGCTGCTCTTCAGATGAT-3’ |
|  | R: 5’- GCGCTGAAGTGCTTTAATGTAT-3’ |
| Mouse *Hemo* (ChIP-qPCR) | F: 5’- CTCCCTTGCACCTGTACCTC-3’ |
|  | R: 5’- CTTTACTGCCCCATGGCTAA-3’ |
| Mouse *Zbtb20*  (Vector contruction) | F: 5’- TCAGGGTACCTTCCTCCAGAAGCCTGTCTA-3’ |
|  | R: 5’- AAAGAGCTCCGGCAACCTCTTGCATTTG-3’ |
| Mouse *Prox1*  (Vector contruction) | F: 5’- TCAGGGTACCTCTAGGCTGATGGCGTTGTGCTG -3’ |
|  | R: 5’- AAAGAGCTCACACTGGCTGCTCTGGAAGGGAA-3’ |
| Mouse *Epha4*  (Vector contruction) | F: 5’- TCAGGGTACCGTGTCTAACTCACTGGGTTCTG-3’ |
|  | R: 5’- AAAGAGCTCCCCTTCCACAAAGGCAACTA-3’ |
| Mouse *Nr4a2*  (Vector contruction) | F: 5’- TCAGGGTACCGAAGCCAGCGGGGCCGGAGAGAGGGAC-3’ |
|  | R: 5’- AAAGAGCTCTGCATGTTGGCAGCAGCAGCTCGA-3’ |
| Mouse *Zbtb20* (qPCR) | F: 5’- CTGTCAGTAACAGCTCCGATAAG-3’ |
|  | R: 5’- GGGTTTCTGTCTGGCGTAAATA-3’ |
| Mouse *Tbr1* (qPCR) | F: 5’- GCTTCGTCACAGTTTCGATGG -3’ |
|  | R: 5’- CCGTTGGTAATGACCGGGTG -3’ |

**Table S6. List of construction carrying point mutation in FOXG1-binding loci**

| Construct name | Primers | Point mutation loci |
| --- | --- | --- |
| Mouse *Zbtb20*  (binding loci mut) | F: 5’- AACCTTTTTGAGGGGGGACAGGCAACA  TGACTCAACAGAATGCT-3’ | G**TA**AACA  G**GC**AACA |
|  | R: 5’-AGCATTCTGTTGAGTCATGTTTGCCTGT  CCCCCCTCAAAAAGGTT-3’ |  |
| Mouse *Zbtb20*  (non-binding loci mut) | F: 5’- TTTTGGAACCTTTTGCGGGGGGACAGTA  AACATGACTCAA-3’ | TTT**TA**G  TTT**GC**G |
|  | R: 5’-TTGAGTCATGTTTACTGTCCCCCCCGAA  AAGGTTCCAAAA-3’ |  |
| Mouse *Prox1*  (binding loci mut) | F: 5’- TAAGGAAGGAGACATGACTCAGCAATAA  GGTGCTGGGAAC-3’ | A**TA**AATA  A**GC**AACA |
|  | R: 5’-GTTCCCAGCACCTTCGTTATGAGTCATGT  CTCCTTCCTTA-3’ |  |
| Mouse *Prox1*  (non-binding loci mut) | F: 5’- CGTTGTGCTGAGGCAGGAAGGAGACATG  ACTCATAAATA-3’ | G**TA**AGG  G**GC**AGG |
|  | R: 5’-TATTTATGAGTCATGTCTCCTTCCTCGCTC  AGCACAACG-3’ |  |
| Mouse *Epha4*  (binding loci mut) | F: 5’- TTCTACTTCCAGTGCAAAGCAACATGCC  ACTTAGAGACAGA-3’ | A**TA**AACA  A**GC**AACA |
|  | R: 5’-TCTGTCTCTAAGTGGCATGTTCGTTTGCA  CTGGAAGTAGAA-3’ |  |
| Mouse *Epha4*  (non-binding loci mut) | F: 5’- AAATAAACATGCCACTGCGAGACAGAC  ATGAAACCACTC-3’ | CT**TA**GAG    CT**GC**GAG |
|  | R: 5’-GAGTGGTTTCATGTCTGTCTCCGAGTGG  CATGTTTATTT-3’ |  |
| Mouse *Nr4a2*  (binding loci mut) | F: 5’- TGGCCCTGGCCGCCAATGTGCCTTTGTT  GCTGTGGCTCGA-3’ | A**TA**AACA  A**GC**AACA |
|  | R: 5’-TCGAGCCACATAAACAAAGGCACATTG  GCGGCCAGGGCCA-3’ |  |
| Mouse *Nr4a2*  (non-binding loci mut) | F: 5’- TGCCTTTGTTTATGTGGCTCGAGCTGCT  GCTGCCAACCGGCA-3’ | C**AT**GCA  C**CG**GCA |
|  | R: 5’-TGCCGGTTGGCAGCAGCAGCTCGAGCC  ACATAAACAAAGGCA-3’ |  |
